# Therapist-assisted online psychological therapies differing in trauma focus for post-traumatic stress disorder (STOP-PTSD): a UK-based, single-blind, randomised controlled trial

**DOI:** 10.1016/S2215-0366(23)00181-5

**Published:** 2023-08

**Authors:** Anke Ehlers, Jennifer Wild, Emma Warnock-Parkes, Nick Grey, Hannah Murray, Alice Kerr, Alexander Rozental, Graham Thew, Magdalena Janecka, Esther T Beierl, Apostolos Tsiachristas, Rafael Perera-Salazar, Gerhard Andersson, David M Clark

**Affiliations:** a Department of Experimental Psychology, University of Oxford, Oxford, UK; b Nuffield Department of Population Health, University of Oxford, Oxford, UK; c Nuffield Department of Primary Care Health Sciences, University of Oxford, Oxford, UK; d Oxford Health NHS Foundation Trust, Oxford, UK; e Phoenix Australia Centre for Posttraumatic Mental Health, Department of Psychiatry, University of Melbourne, Melbourne, VIC, Australia; f Department of Psychology, Institute of Psychiatry, Psychology & Neuroscience, King's College London, London, UK; g South London and Maudsley NHS Foundation Trust, London, UK; h Sussex Partnership NHS Foundation Trust, Worthing, UK; i Department of Psychology, Uppsala University, Uppsala, Sweden; j Centre for Psychiatry Research, Department of Clinical Neuroscience, Karolinska Institutet, Stockholm, Sweden; k Icahn School of Medicine at Mount Sinai, New York City, NY, USA; l Department of Behavioural Sciences and Learning and Department of Biomedical and Clinical Sciences, Linköping University, Linköping, Sweden

## Abstract

**Background:**

Many patients are currently unable to access psychological treatments for post-traumatic stress disorder (PTSD), and it is unclear which types of therapist-assisted internet-based treatments work best. We aimed to investigate whether a novel internet-delivered cognitive therapy for PTSD (iCT-PTSD), which implements all procedures of a first-line, trauma-focused intervention recommended by the UK National Institute for Health and Care Excellence (NICE) for PTSD, is superior to internet-delivered stress management therapy for PTSD (iStress-PTSD), a comprehensive cognitive behavioural treatment programme focusing on a wide range of coping skills.

**Methods:**

We did a single-blind, randomised controlled trial in three locations in the UK. Participants (≥18 years) were recruited from UK National Health Service (NHS) Improving Access to Psychological Therapies (IAPT) services or by self-referral and met DSM-5 criteria for PTSD to single or multiple events. Participants were randomly allocated by a computer programme (3:3:1) to iCT-PTSD, iStress-PTSD, or a 3-month waiting list with usual NHS care, after which patients who still met PTSD criteria were randomly allocated (1:1) to iCT-PTSD or iStress-PTSD. Randomisation was stratified by location, duration of PTSD (<18 months or ≥18 months), and severity of PTSD symptoms (high *vs* low). iCT-PTSD and iStress-PTSD were delivered online with therapist support by messages and short weekly phone calls over the first 12 weeks (weekly treatment phase), and three phone calls over the next 3 months (booster phase). The primary outcome was the severity of PTSD symptoms at 13 weeks after random assignment, measured by self-report on the PTSD Checklist for DSM-5 (PCL-5), and analysed by intention-to-treat. Safety was assessed in all participants who started treatment. Process analyses investigated acceptability and compliance with treatment, and candidate moderators and mediators of outcome. The trial was prospectively registered with the ISRCTN registry, ISRCTN16806208.

**Findings:**

Of the 217 participants, 158 (73%) self-reported as female, 57 (26%) as male, and two (1%) as other; 170 (78%) were White British, 20 (9%) were other White, six (3%) were Asian, ten (5%) were Black, eight (4%) had a mixed ethnic background, and three (1%) had other ethnic backgrounds. Mean age was 36·36 years (SD 12·11; range 18–71 years). 52 (24%) participants met self-reported criteria for ICD-11 complex PTSD. Fewer than 10% of participants dropped out of each treatment group. iCT-PTSD was superior to iStress-PTSD in reducing PTSD symptoms, showing an adjusted difference on the PCL-5 of –4·92 (95% CI –8·92 to –0·92; p=0·016; standardised effect size *d*=0·38 [0·07 to 0·69]) for immediate allocations and –5·82 (–9·59 to –2·04; p=0·0027; *d*=0·44 [0·15 to 0·72]) for all treatment allocations. Both treatments were superior to the waiting list for PCL-5 at 13 weeks (*d*=1·67 [1·23 to 2·10] for iCT-PTSD and 1·29 [0·85 to 1·72] for iStress-PTSD). The advantages in outcome for iCT-PTSD were greater for participants with high dissociation or complex PTSD symptoms, and mediation analyses showed both treatments worked by changing negative meanings of the trauma, unhelpful coping, and flashback memories. No serious adverse events were reported.

**Interpretation:**

Trauma-focused iCT-PTSD is effective and acceptable to patients with PTSD, and superior to a non-trauma-focused cognitive behavioural stress management therapy, suggesting that iCT-PTSD is an effective way of delivering the contents of CT-PTSD, one of the NICE-recommended first-line treatments for PTSD, while reducing therapist time compared with face-to-face therapy.

**Funding:**

Wellcome Trust, UK National Institute for Health and Care Research Oxford Health Biomedical Research Centre.

## Introduction

The UK National Institute for Health and Care Excellence (NICE)^1^ and international treatment guidelines^2^ recommend trauma-focused psychological therapies, including several forms of trauma-focused cognitive behavioural therapy (CBT), as first-line treatments for post-traumatic stress disorder (PTSD). A 2013 Cochrane review,^3^ however, suggested that non-trauma-focused CBT could be similarly effective. Direct comparisons to evaluate the relative merits of trauma-focused CBT (eg, cognitive therapy for PTSD,^4^ one of the first-line treatments for PTSD recommended by NICE^1^) and non-trauma-focused CBT (eg, stress-management therapy^5^) in the treatment of PTSD are warranted.

**Research in context**

**Evidence before this study**

Face-to-face, individual, trauma-focused cognitive behaviour therapy (CBT) has been shown to be highly effective in randomised controlled trials, and evidence supports it as a first-line treatment for post-traumatic stress disorder (PTSD), although face-to-face non-trauma-focused CBT has also shown good outcomes in the short term. However, many people with PTSD cannot access these treatments due to a shortage of trained therapists, their geographical location, or being unable to attend during usual office hours. Internet-delivered CBT (iCBT) could help to improve access and reduce therapist time. Several iCBT programmes for PTSD implement some of the trauma-focused CBT procedures, and a 2021 Cochrane review found that these programmes lead to moderately better outcomes than waiting lists (*d*=0·61) but are not superior to other treatments. However, there were large differences in effect sizes between studies, with some suggesting that outcomes similar to face-to-face therapy can be attained with specific trauma-focused iCBT programmes. One programme (Spring) has been shown to be non-inferior to face-to-face therapy at 16 weeks but not at 52-week follow-up. There is a need to investigate which types of iCBT treatments work best, and especially whether trauma-focused iCBT works better than non-trauma-focused iCBT. We searched PubMed on Feb 2, 2023, with the terms ((“randomised”) OR (“randomized”) OR (“trial”)) AND ((“PTSD”) OR (“posttraumatic”) OR (“post-traumatic”)) AND ((“internet”) OR (“digital”)) AND ((“CBT”) OR (“therapy”)) with no language restrictions. We identified 153 records, including five relevant RCTs published since the 2021 Cochrane review, that compared different versions of iCBT for PTSD with waiting lists or face-to-face therapy. No studies compared a trauma-focused with a non-trauma-focused iCBT programme in patients with PTSD.

**Added value of this study**

To our knowledge, this is the first study directly comparing a trauma-focused therapist-assisted iCBT programme with a comprehensive non-trauma-focused iCBT programme in people with PTSD. iCT-PTSD, which faithfully implements all the procedures of the corresponding first-line treatments for PTSD recommended by UK National Institute for Health and Care Excellence (NICE), and iStress-PTSD, a comprehensive programme focusing on strategies for coping with stress and PTSD symptoms were both acceptable and credible to patients and were efficacious compared with waiting list plus usual UK National Health Service (NHS) care. iCT-PTSD was superior to an internet-based stress management therapy for PTSD (iStress-PTSD) in symptoms of PTSD and other outcomes including quality of life. Both treatments were shown to work by changing negative meanings of the trauma, unhelpful coping, and flashback memories. iCT-PTSD achieved comparable recovery rates to those found for face-to-face CT-PTSD in previous randomised trials and better recovery rates (77%) than currently observed in Improving Access to Psychological Therapies services (41% for 2021–22).

**Implications of all the available evidence**

The superiority of trauma-focused iCBT-PTSD to the non-trauma-focused iCBT-PTSD suggests that specific work on trauma memories, their meanings, and their triggers has added benefits compared to good generic non-trauma-focused iCBT. We found that iStress-PTSD participants who chose trauma-related hierarchies for their exposure practice had better outcomes, which is in line with the added benefits of trauma-focused work. iCT-PTSD is an efficacious treatment that could help to make a first-line treatment recommended by NICE more widely accessible to patients, save more than half of the therapist's time, and help improve recovery rates in routine clinical care, such as NHS Talking Therapies for Anxiety and Depression (formerly Improving Access to Psychological Therapies) services, through consistent delivery of all core treatment procedures.

Many people with PTSD are unable to access effective psychological treatments for a range of reasons, such as a shortage of therapists or being unable to attend therapy during usual working hours. It is therefore desirable to develop efficient forms of treatment delivery that can be more easily accessed than currently. Therapist-assisted, internet-based treatment delivery appears to be a promising option. There is evidence that therapist-assisted, internet-based psychological treatments are effective for PTSD. A 2021 Cochrane review^6^ found that internet-based CBT (iCBT) was superior to a waiting list for reducing PTSD symptoms, with a standardised mean difference of 0·61, but there were large differences in effect sizes between different programmes, with some therapist-assisted programmes^7^, ^8^ showing large to very large effect sizes. That review did not find differences between iCBT and other internet-based treatments. A multisite effectiveness study^9^ found that a trauma-focused, guided self-help iCBT programme (Spring) was non-inferior to face-to-face trauma-focused CBT in people with mild to moderate PTSD at 16-week follow-up but not at 52-week follow-up.

It remains unclear which types of internet-based treatments for PTSD work best and are most acceptable to patients, and whether a trauma-focused approach is advantageous for internet-based treatment. This study compared a novel trauma-focused, therapist-assisted online psychological therapy (internet-based cognitive therapy for PTSD [iCT-PTSD]^10^) with a non-trauma-focused, therapist-assisted online psychological therapy (internet-based stress management therapy for PTSD [iStress-PTSD]^5^). iCT-PTSD is an online version of trauma-focused cognitive therapy for PTSD.^4^, ^11^ A consecutive case series evaluation of iCT-PTSD^10^ suggested that it could be as effective as face-to-face cognitive therapy for PTSD. iStress-PTSD is based on a comprehensive, internet-based, CBT stress-management programme developed by Andersson and colleagues,^5^ which includes applied relaxation, mindfulness, thought challenging, and exposure to avoided situations, and has been shown to be effective in several randomised trials.^5^, ^12^ The programme was translated into English and adapted for patients with PTSD by HM. Both treatments were compared with a waiting list plus usual UK National Health Service (NHS) care to control for the natural recovery that is sometimes seen in people with PTSD.^13^

We aimed to assess whether iCT-PTSD was superior to iStress-PTSD in reducing symptoms of PTSD; whether iCT-PTSD and iStress-PTSD are efficacious in leading to greater reduction in PTSD symptoms than being on a waiting list while receiving usual NHS care. We also aimed to assess whether iCT-PTSD leads to greater improvement in depression, anxiety, wellbeing, disability, quality of life, and sleep problems than iStress-PTSD, and whether iCT-PTSD and iStress-PTSD lead to greater improvements in those outcomes than the waiting list control. Through process analyses, we also aimed to investigate the acceptability and compliance with the treatments among participants, any adverse effects, and moderators and mediators of outcome.

## Methods

### Study design and participants

We did a single-blind randomised controlled superiority trial with an embedded process study in three locations in the UK (Thames Valley, London, and Sussex). Participants were mainly recruited from Improving Access to Psychological Therapies (IAPT) services in rural and urban areas (Buckinghamshire, Berkshire, Croydon, Lambeth, Lewisham, Oxfordshire, Southwark, Brighton and Hove, and East Sussex). It should be noted that in 2023, NHS England renamed IAPT as NHS Talking Therapies for Anxiety and Depression. Participants in the same areas could also self-refer in response to information listed on study and trial registration websites (ISRCTN; UK Clinical Trials Gateway).

Eligibility criteria were chosen to recruit people with a wide range of PTSD severity (appendix p 2).^14^ The eligibility assessment included the Structured Clinical Interview for DSM-5 (SCID)^15^, ^16^ with a clinician, to assess PTSD and comorbid axis I and axis II disorders. Comorbidities (eg, comorbid depression, other anxiety disorders, or substance misuse), a history of previous trauma (eg, childhood abuse), and previous treatment for PTSD are common in people with PTSD and were not exclusion criteria. Participants were 18 years or older, met diagnostic criteria for PTSD as assessed through the SCID,^15^ had PTSD as their main diagnosis (ie, their main clinical problem that required treatment at present as decided in the clinical assessment with a clinician), were able to read and write in English, and had access to the internet. Exclusion criteria were a history of psychosis, current substance dependence, current borderline personality disorder, and acute serious suicide risk. If taking psychotropic medication, participants were required to be on a stable dose for at least 1 month before random assignment and were asked to maintain this dose during treatment. If currently receiving psychological therapy for PTSD at the time of recruitment, this treatment had to end before random assignment.

The trial protocol has been published^14^ and is available online. The study had NHS Research Ethics approval (West Midland–The Black Country Research Ethics Committee, 17/WM/0441; IRAS 224759), and a Trial Oversight Committee, comprising experts and a service user, reviewed the protocol and statistical analysis plan and monitored progress.

### Procedures

iCT-PTSD and iStress-PTSD were delivered online via a series of therapy modules with therapist support by messages within the online programme, SMS, and short weekly phone calls (designed to last on average 20 min) over the first 12 weeks (weekly treatment phase), and three monthly phone calls over the next 3 months (booster phase). Therapists could, with the participant's knowledge, read the information that they provided in the modules and write notes for them directly into the modules.

The therapists were clinical psychologists experienced in providing face-to-face CBT. Therapists received training in the delivery of both online treatments and treated at least one person as a supervised training case with each treatment. Training involved familiarisation with the content of modules and the functionality of the programme and group supervision. There was no specified timeframe for training. Therapists received weekly group supervision for each of the treatment types to ensure adherence to protocol and to ensure high quality of treatment delivery. iCT-PTSD supervision was led by AE (expert in CT-PTSD), and iStress-PTSD supervision was led by AR (expert in stress management therapy). Therapists’ adherence to treatment components was assessed by independent raters (graduate psychologists) from a randomly selected audio recording of a whole phone call for each participant and the messages therapists sent via the online system for the week of this call. Treatment credibility (participant and therapist ratings)^17^ and working alliance^18^ were assessed by self-report questionnaires in week 2 of treatment. Participants’ compliance with treatment was assessed by them completing therapy, time spent on the programme, and percentage of module completion.

iCT-PTSD implements all procedures of cognitive therapy for PTSD, one of the trauma-focused CBT programmes recommended by NICE and international treatment guidelines.^1^, ^2^ The treatment builds on Ehlers’ and Clark's model of PTSD^19^ and focuses on changing problematic appraisals of trauma and its aftermath, which induce a sense of current threat, as well as on updating trauma memories, identifying and discriminating triggers of re-experiencing symptoms, and changing unhelpful behaviours that maintain the symptoms, and prevent changes in appraisals and memory features. iCT-PTSD does not include iStress-PTSD's training in stress-reduction strategies (eg, applied relaxation and mindfulness).

iStress is a stress-management therapy programme focusing on learning and practising a wide range of coping skills that have been shown to be efficacious with diverse groups experiencing stressors.^5^, ^12^ iStress was adapted for people with PTSD by HM (ie, the content was adapted for PTSD, and additional modules were written; adaptations were approved by GA). iStress-PTSD includes psychoeducation about PTSD, training in problem solving, applied relaxation training, challenging irrational thoughts, mindfulness, improving sleep efficiency, and in vivo exposure to avoided situations. Participants also work on challenging areas of their choice, such as coping with memories or worry. They choose the areas of stress to which they apply the techniques with support from their therapist. The programme does not include iCT-PTSD's specific trauma-focused procedures for testing trauma-related appraisals or working on the content of trauma memories and their triggers. Further details are in the appendix (pp 4–8).

Waiting list with usual clinical care involved 3 months of usual clinical care (general practitioner and other treating NHS services, eg, a stable dose of psychotropic medication, and treatment for pain and comorbid medical problems) while waiting for allocation to one of the internet-delivered treatments. Participants completed assessments at baseline (ie, at randomisation) and at 6 weeks and 13 weeks after randomisation. If participants no longer met PTSD criteria at 13 weeks, their participation in the trial was finished and they were offered treatment for residual symptoms.

### Randomisation and masking

Eligible participants were randomly assigned (3:3:1) to iCT-PTSD, iStress-PTSD, or waiting list plus usual care, stratified by location (Thames Valley, London, or Sussex), duration of PTSD (<18 months or ≥18 months), and severity of PTSD symptoms on the PTSD Checklist for DSM-5 ([PCL-5]; high [≥49] *vs* low [<49]), using an online random allocation programme (Sortition) developed for this study by the Primary Care Clinical Trials Unit at the University of Oxford. The programme uses a minimisation algorithm with a random component. The allocation sequence was not visible to the administrators who generated the treatment allocation with the programme. Participants originally allocated to the waiting list control who had not recovered from PTSD at 13 weeks were randomly assigned (1:1) to iCT-PTSD or iStress-PTSD.

Independent assessors conducting Clinician-Administered PTSD Scale (CAPS-5) interviews were masked to study group assignment, and participants were reminded not to disclose the trial condition to which they were assigned. If participants accidentally mentioned information that unmasked the assessor, this information was cut from the recording, which was then re-rated by another rater. Therapists, trial administrators, and participants were not masked to treatment allocation due to the nature of the intervention. Statistical analyses were done masked to treatment, with the treatments coded as A and B.

### Outcomes

Assessments occurred at 6 weeks, 13 weeks (end of weekly phase or waiting list), 26 weeks (end of booster phase), 39 weeks, and 65 weeks after randomisation. The primary outcome was PTSD symptom severity on the self-reported PCL-5 at 13 weeks.^20^ Secondary outcomes included two further measures of PTSD symptom severity to aid comparison with other studies: the Impact of Event Scale-Revised,^21^ which was the PTSD measure used by IAPT Services, and the CAPS-5.^22^ Inter-rater reliability for the CAPS-5 was assessed for PTSD diagnosis and severity by obtaining a second rating for each item from another independent assessor.

Secondary measures of other outcomes included the Patient Health Questionnaire (PHQ-9)^23^ for symptoms of depression; the Generalized Anxiety Disorder Scale (GAD-7)^24^ for symptoms of anxiety; the Work and Social Adjustment Scale (WSAS)^25^ for disability and interference with functioning; the WHO-5 Well-Being Index^26^ for psychological wellbeing; the Quality of Life Enjoyment and Satisfaction Scale (Q-LES-Q)^27^ to assess general quality of life; and the Insomnia Sleep Index (ISI)^28^ for sleep disturbance. We also report measures from the EuroQol 5 dimensions 5 levels questionnaire (EQ-5D-5L),^29^ a standard measure of health-related quality of life. A full health economic analysis will be reported separately.

The IAPT Patient Experience Questionnaire^30^ assessed patient's satisfaction with treatment at 13 weeks. Qualitative interviews will be reported elsewhere. Further measures included measures of psychological processes used in the mediation analyses (appendix p 35), and measures for missing data or moderation analyses.^31^, ^32^, ^33^, ^34^ Demographic information such as gender (male, female, or other) and age, clinical characteristics such as dissociation, substance use and trauma history were assessed by self-report questionnaires. Further information on traumas and clinical and treatment history was assessed by a clinician together with the SCID. Complex PTSD was assessed according to ICD-11 (using the International Trauma Questionnaire).

### Choice of primary outcome

The PCL-5 scale^20^ was chosen a priori^14^ for the primary outcome measure because it covers all PTSD symptoms defined in DSM-5, is a widely used self-report measure for PTSD, takes only 5 min to complete, and is freely available in several languages. It was chosen to maximise data completion and for direct comparability to published national IAPT data, which are also based on self-reports. Compared with the CAPS-5, the PCL-5 is less time-consuming and does not involve having to talk about distressing symptoms with a person the participant has potentially not met before. In our experience, the PCL-5 therefore tends to ensure higher response rates at the end of treatment and follow-ups than CAPS-5 does, especially for participants who did not benefit much from treatment, which reduces bias towards positive outcomes in completed data. A 5-point difference on the PCL-5 is commonly considered the minimum difference^20^ between treatment groups to be clinically meaningful.^14^ The 13-week assessment after the end of the weekly phone calls in the treatment groups and the waiting list control group was chosen as the primary assessment point, whereas the 26-week assessment represented the end of all treatment after the booster treatment phase with ongoing work on the online modules and monthly phone calls.

### Statistical analysis

We aimed to recruit 217 participants (93 per treatment group and 31 for the waiting list) to have 80% power to detect an effect size of Cohen's *d* coefficient of 0·50 between iCT-PTSD and iStress-PTSD, allowing for 15% dropouts, an average cluster size of 12, and a coefficient of variation of 0·68.^14^ This effect size corresponds to the threshold set by NICE for clinically significant differences between medication and placebo in PTSD,^35^ and to a clinically meaningful difference on the PCL-5 (ie, 5–6 points for SDs of 10–12).

Statistical analyses were pre-specified in the statistical analysis plan before the end of the trial and then followed (appendix p 37). The trial protocol and statistical analysis plan were discussed and agreed with the Trial Oversight Committee. Continuous outcomes were analysed using linear mixed effects regression models, which use all available data from randomly assigned participants (intention-to-treat analysis), can account for repeated measures, and implicitly account for data missing at random. Models of treatment effects included categorical fixed factors of time (6 weeks, 13 weeks, and 26 weeks after randomisation), treatment (iCT-PTSD and iStress-PTSD), and the time by treatment interaction. The interaction allows the estimation of differences between treatments at each timepoint. The stratification variables site, time since trauma, and baseline PCL-5 score were included as fixed covariates, along with the baseline score on the measure being analysed. Participant was specified as a random effect to account for between-participant variation. Models examining the maintenance of treatment effects used scores at the end of treatment (26 weeks or 13 weeks if missing) and follow-ups at 39 weeks and 65 weeks. The significance level was set as p values less than 0·05 for comparisons between iCT-PTSD and iStress-PTSD, and was adjusted to p values less than 0·025 for comparisons of each of the treatments with the waiting list condition. Analyses were performed in R (version 4.0.3) using the tidyverse, nlme, and psych packages.

All models used restricted maximum likelihood estimation and an unstructured covariance matrix. Q–Q plots indicated that the normality of residuals assumption was met for all models. Standardised between-group effect sizes (*d*) were calculated by dividing the adjusted group difference by the baseline standard deviation. Standardised within-group effect sizes (*d*) were calculated from separate models that incorporated the baseline score as a timepoint rather than as a covariate, to obtain within-group adjusted means in relation to baseline.

The primary analysis compared participants who were immediately allocated to iCT-PTSD and iStress-PTSD on PCL-5 scores at 13 weeks. Several sensitivity analyses were performed. First, we explored variables related to missing data in the primary outcome at 13 weeks, including any significant predictors in the analysis. Second, we did multilevel multiple imputation of missing data with the mice R package, using all available baseline variables. Third, only participants who had received at least a minimum dose of therapy that can be expected to have clinical benefits were analysed, defined as completing core procedures relating to (1) rationale and psychoeducation, (2) activities in everyday life, and (3) at least one core technique addressing trauma memories in iCT-PTSD or one core stress-management technique in iStress-PTSD, respectively (appendix p 23). Fourth, we included all participants who were randomly allocated to one of the treatments, including post-waiting list allocations. As pre-specified in the statistical analysis plan, further analyses used all randomly assigned participants. A complier-average casual-effect analysis is presented in the appendix (p 23).

Further linear mixed-effects regression models analyses tested whether any demographic, clinical, or trauma characteristics influenced the size of the difference in the primary outcome between the treatments (moderator analyses; appendix p 25) and whether differences in outcome between the treatments and waiting list control, and between iCT-PTSD and iStress-PTSD were mediated by cognitive factors hypothesised to maintain PTSD^19^ (mediation analyses; appendix p 27). A planned exploratory analysis examined whether the degree of trauma-focused exposure undertaken within iStress-PTSD was associated with clinical outcomes on the primary outcome measure (PCL-5) at 13 weeks and 26 weeks (appendix p 31). The trial was prospectively registered with the ISCRCTN registry, ISRCTN16806208.

### Role of the funding source

The funders of the study were not involved in designing the study, data collection, data analysis, data interpretation, writing of reports, or the decision to submit the manuscript for publication.

## Results

**Figure 1:**
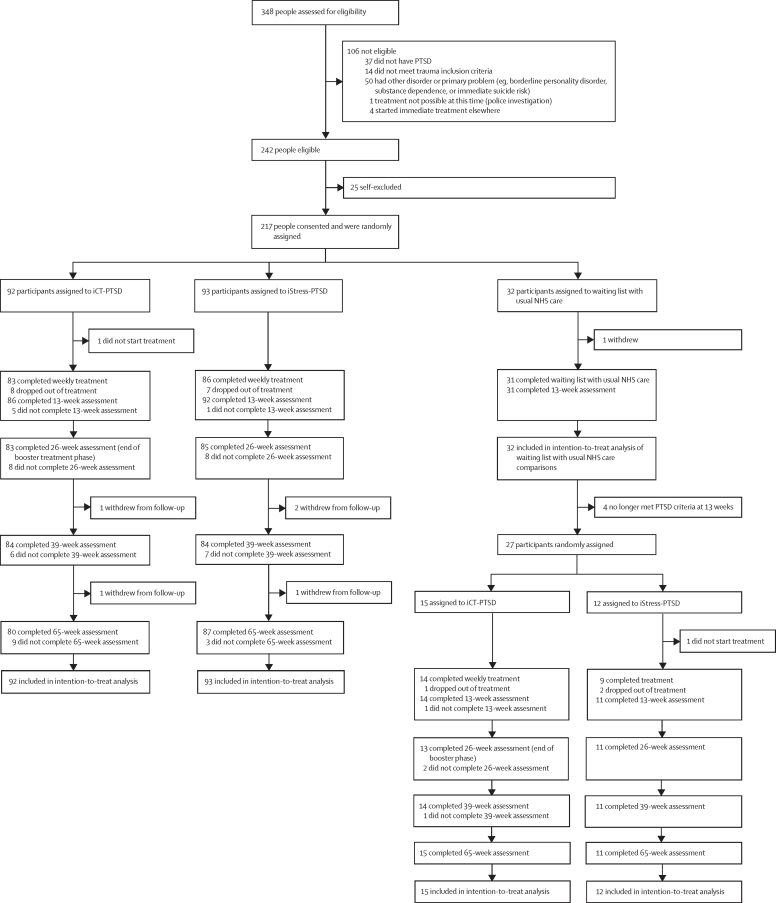
CONSORT flow diagram iCT-PTSD=internet-delivered cognitive therapy for PTSD. iStress-PTSD=internet-based stress management therapy for PTSD. NHS=National Health Service. PTSD=post-traumatic stress disorder.

Participants were recruited between Jan 15, 2018, and March 31, 2020. 348 patients were assessed for eligibility, of whom 242 were eligible and 217 consented to participate. Of those 217 participants, 92 (42%) were randomly assigned to iCT-PTSD, 93 (43%) were randomly assigned to iStress-PTSD, and 32 (15%) were randomly assigned to waiting list with usual care (figure 1). After the waiting list period, four participants no longer met the full criteria for PTSD, and the remaining 27 participants were randomly allocated to iCT-PTSD (n=15) or iStress-PTSD (n=12). Only one participant in each group did not start treatment or the waiting list control. Including allocations after the waiting list period, ten (9%) of 107 participants in the iCT-PTSD group and ten (10%) of 105 participants in the iStress-PTSD group did not start or dropped out of treatment, and nine (8%) participants in each treatment group did not receive the minimum dose of therapy. Three (3%) patients in each treatment group withdrew during follow-up (appendix p 9). Data for the primary outcome for the comparison between iCT-PTSD and iStress-PTSD were available for 203 (96%) of 212 participants assigned to treatment at 13 weeks, 192 (91%) at 26 weeks, 193 (91%) at 39 weeks, and 193 (91%) at 65 weeks.

Of the 217 participants who were randomly assigned, 158 (73%) were female, 57 (26%) were male, and two (1%) had another gender; 170 (78%) were White (British), 20 (9%) were White (other), six were Asian (3%), ten (5%) were Black, eight (4%) had a mixed ethnic background, and three (1%) had another ethnic background (table 1).^36^ The mean age of participants was 36·36 years (SD 12·11; range 18–71). Participants had experienced an average of just under five types of trauma in their lifetimes, and treatment addressed between one and four traumas. The mean time since the main traumas that were addressed in treatment happened was 3·5 years (6·3). Traumas included sexual violence (35 [16%] of 217 participants); physical violence (32 [15%]); death, severe illness, or harm to significant other (41 [19%]); medical trauma including traumatic childbirth (35 [16%]); accidents or natural disasters (53 [24%]); and combat or professionals witnessing harm to others (21 [10%]). 29 (13%) of 217 participants had a permanent physical disability due to the trauma, 52 (24%) met the self-report criteria for complex PTSD,^32^ 177 (82%) had at least one comorbid mental disorder, 60 (24%) reported a history of self-harm, and 19 (9%) reported a history of one or more suicide attempts.

**Table 1:** Demographics and trauma characteristics

			**iCT-PTSD (n=92)**	**iStress-PTSD (n=93)**	**Waiting list with usual NHS care (n=32)**	**Total (n=217)**
Age, years			36·25 (12·21)	35·80 (11·46)	38·32 (13·79)	36·36 (12·11)
Gender						
	Female		68 (74%)	68 (73%)	22 (69%)	158 (73%)
	Male		24 (26%)	23 (25%)	10 (31%)	57 (26%)
	Other		0	2 (2%)	0 (0%)	2 (1%)
Race or ethnicity						
	White (British)		68 (74%)	76 (82%)	26 (81%)	170 (78%)
	White (other)		12 (13%)	4 (4%)	4 (13%)	20 (9%)
	Asian		2 (2%)	3 (3%)	1 (3%)	6 (3%)
	Black		4 (4%)	6 (6%)	0 (0%)	10 (5%)
	Mixed ethnic background		3 (3%)	4 (4%)	1 (3%)	8 (4%)
	Other ethnic background		3 (3%)	0	0	3 (1%)
Religion						
	Christian		39/83 (47%)	36/88 (41%)	13/29 (45%)	88/200 (44%)
	Other religion		4/83 (4%)	4/88 (4%)	2/29 (7%)	10/200 (5%)
	None		35/83 (43%)	43/88 (49%)	11/29 (38%)	89/200 (45%)
	Other		0	3/88 (3%)	1/29 (3%)	4/200 (2%)
	Do not wish to specify		5/83 (6%)	2/88 (2%)	2/29 (7%)	9/200 (5%)
	Missing		9 (1%)	5 (<1%)	3 (1%)	17 (1%)
Level of education						
	None completed		1/90 (1%)	3/90 (3%)	1/29 (3%)	5/209 (2%)
	School qualification at age 16 years		17/90 (19%)	23/90 (26%)	7/29 (24%)	47/209 (22%)
	School qualification at age 18 years		20/90 (22%)	15/90 (17%)	5/29 (17%)	40/209 (19%)
	Professional qualification		16/90 (18%)	6/90 (7%)	3/29 (10%)	25/209 (12%)
	University graduate		36/90 (40%)	43/90 (48%)	13/29 (45%)	92/209 (44%)
	Missing		2 (2%)	3 (3%)	3 (9%)	8 (4%)
Marital status						
	Single		37 (40%)	35 (38%)	12 (38%)	84 (39%)
	Married		29 (32%)	33 (35%)	15 (47%)	77 (35%)
	Cohabitating with partner		18 (20%)	15 (16%)	3 (9%)	36 (17%)
	Divorced or separated		6 (7%)	9 (10%)	1 (3%)	16 (7%)
	Widowed		2 (2%)	1 (1%)	1 (3%)	4 (2%)
Months since main trauma			48·78 (84·74)	37·22 (67·68)	32·59 (71·56)	41·44 (75·85)
Reported serious social or financial problems						
	No		64/91 (70%)	65/90 (72%)	18 (56%)	147/213 (69%)
	Yes		27/91 (30%)	25/90 (28%)	14 (44%)	66/213 (31%)
	Missing		1 (1%)	3 (3%)	0	4 (2%)
Employment status						
	Full-time		45 (49%)	41 (44%)	13 (41%)	99 (46%)
	Part-time		17 (18%)	12 (13%)	8 (25%)	37 (17%)
	Self-employed		5 (5%)	10 (11%)	3 (9%)	18 (8%)
	Unemployed (seeking work)		1 (1%)	1 (1%)	0 (0%)	2 (1%)
	Long-term unemployed		2 (2%)	2 (2%)	2 (6%)	6 (3%)
	Full-time student		10 (11%)	7 (8%)	1 (3%)	18 (8%)
	Part-time student		1 (1%)	0 (0%)	1 (3%)	2 (1%)
	Long-term sick leave or disability benefits		5 (5%)	7 (8%)	1 (3%)	13 (6%)
	Full-time homemaker or carer		2 (2%)	8 (9%)	1 (3%)	11 (5%)
	Unpaid volunteer work		1 (1%)	1 (1%)	0 (0%)	2 (1%)
	Retired		3 (3%)	3 (3%)	2 (6%)	8 (4%)
	Rather not say		0	1 (1%)	0	1 (<1%)
Household income per year, GBP£						
	≤15 000		15/81 (19%)	16/87 (18%)	5/28 (18%)	36/205 (18%)
	15 001–25 000		18/81 (22%)	12/87 (14%)	4/28 (14%)	34/205 (17%)
	25 001–35 000		8/81 (10%)	22/87 (25%)	5/28 (18%)	35/205 (18%)
	35 001–50 000		14/81 (17%)	15/87 (17%)	5/28 (18%)	34/205 (17%)
	50 001–70 000		13/81 (16%)	5/87 (6%)	3/28 (11%)	21/205 (11%)
	>70 000		13/81 (16%)	17/87 (20%)	6/28 (21%)	36/205 (18%)
	Missing		11 (12%)	6 (6%)	4 (13%)	21 (10%)
Disability						
	Reported at least one disability		17 (19%)	16 (17%)	3 (13%)	37 (17%)
Treatment history and comorbidity						
	Taking psychotropic medication		41 (45%)	37 (40%)	12 (38%)	90 (41%)
	History of psychological therapy					
		No	38 (41%)	35/92 (38%)	16 (50%)	89/216 (41%)
		Yes	54 (59%)	57/92 (62%)	16 (50%)	127/216 (59%)
		Missing	0	1 (1%)	0	1 (>1%)
	History of treatment for PTSD					
		No	79/91 (87%)	86 (92%)	31 (97%)	196 (91%)
		Yes	12/91 (13%)	7 (8%)	1 (3%)	20 (9%)
		Missing	1 (1%)	0	0	1 (<1%)
	Meets self-reported ICD-11 criteria for complex PTSD (ITQ^32^)		22 (24%)	24 (26%)	6 (19%)	52 (24%)
	High levels of dissociation (TSDQ^36^)		27 (29%)	22 (24%)	7 (22%)	56 (26%)
	Physical problems that currently require treatment					
		No	57/86 (66%)	63/87 (72%)	22/30 (73%)	142/203 (70%)
		Yes	29/86 (34%)	24/87 (28%)	8/30 (27%)	61/203 (30%)
		Missing	6 (7%)	6 (6%)	2 (6%)	14 (6%)
	Current anxiety disorder (SCID^15^)		53 (58%)	61 (66%)	20 (62%)	134 (62%)
	Current depressive disorder (SCID^15^)		57 (62%)	59 (63%)	11 (34%)	127 (59%)
	History of major depression (SCID^15^)		23 (25%)	26 (28%)	11 (34%)	60 (28%)
	Current OCD–related disorder (SCID^15^)		13 (14%)	8 (9%)	3 (9%)	24 (11%)
	Current somatoform disorder (SCID^15^)		3 (3%)	6 (7%)	0 (0%)	9 (4%)
	Current substance misuse (SCID^15^)		2 (2%)	2 (2%)	3 (10%)	7 (3%)
	High substance use (AUDIT^34^)		31 (34%)	39 (42%)	13 (41%)	83 (38%)
	Current eating disorder (SCID^15^)		2 (2%)	3 (3%)	4 (12%)	9 (4%)
	Any current comorbid mental disorder		75 (82%)	76/93 (82%)	26/31 (81%)	177/215 (82%)
		Missing	0	1 (1%)	1 (3%)	2 (1%)
	Screens positive for personality disorder (SAPAS^33^)					
		No	56 (61%)	59/92 (64%)	21/31 (68%)	136/215 (63%)
		Yes	36 (39%)	33/92 (36%)	10/31 (32%)	79/215 (37%)
		Missing	0	1 (1%)	1 (3%)	2 (1%)
	History of self-harm		23 (25%)	28 (30%)	9 (28%)	60 (28%)
	History of one or more suicide attempts		10 (11%)	4 (4%)	5 (16%)	19 (9%)
Information on trauma						
	Type of main trauma					
		Sexual violence	17 (18%)	15 (16%)	3 (9%)	35 (16%)
		Physical violence	12 (13%)	15 (16%)	5 (16%)	32 (15%)
		Death, severe illness, or harm of significant other	17 (18%)	22 (24%)	2 (6%)	41 (19%)
		Combat or harm to others	9 (10%)	10 (11%)	2 (6%)	21 (10%)
		Accident or natural disaster	27 (29%)	16 (17%)	10 (31%)	53 (24%)
		Medical trauma or childbirth	10 (11%)	15 (16%)	10 (31%)	35 (16%)
	Numbers of traumas addressed in treatment					
		1	75 (81%)	78 (84%)	27 (84%)	180 (83%)
		2–4	17 (19%)	15 (16%)	5 (16%)	37 (17%)
	Permanent physical disability due to trauma					
		None	79 (86%)	83 (89%)	26 (81%)	188 (87%)
		Moderate	9 (10%)	6 (7%)	2 (6%)	17 (8%)
		Severe or life–changing	4 (4%)	4 (4%)	4 (12%)	12 (6%)
	Number of trauma types experienced (LEC^31^)		5·09 (3·26)	4·74 (3·02)	4·45 (3·58)	4·85 (3·20)
		Missing	0	0	1	1
	History of neglect in childhood		7 (7·6%)	9 (9·7%)	2 (6·2%)	18 (8%)
	History of childhood sexual abuse		13 (14%)	10 (11%)	4 (12%)	27 (12%)
	History of childhood physical abuse		16 (17%)	15 (16%)	3 (9·4%)	34 (16%)
	History of any childhood abuse		25 (27%)	23 (25%)	6 (19%)	54 (25%)

Data are mean (SD) or n (%). AUDIT=Alcohol Use Disorders Identification Test. iCT-PTSD=internet-delivered cognitive therapy for PTSD. iStress-PTSD=internet-based stress management therapy for PTSD. ITQ=international trauma questionnaire. LEC=Life Events Checklist. NHS=National Health Service. OCD=obsessive-compulsive disorder. PTSD=post–traumatic stress disorder. SAPAS=Structured Assessment of Personality Abbreviated Scale. SCID=Structured Clinical Interview for DSM-5. TSDQ=Trait-State Dissociation Questionnaire.

Treatment fidelity was excellent, with only two minor deviations identified by the raters across recordings from 207 participants, both of which concerned using specific cognitive therapy techniques in the iStress-PTSD condition. Therapist competence within phone calls and messages was rated as high (on a scale of 0 to 6) for both iCT-PTSD (mean 5·49 [SD 0·49], n=104) and iStress-PTSD (mean 5·49 [SD 0·43], n=103) groups. Participant compliance was high, and ratings of treatment credibility^18^ and working alliance ratings^19^ by participants and therapists were very high and did not differ between treatment groups (appendix p 11). 28 CAPS-5 interviews were re-rated because participants accidentally mentioned information that unmasked the assessors, which was removed from the recordings.Inter-rater reliability for the CAPS-5 was high (intraclass correlation 0·88 for PTSD diagnosis, and 0·95 for PTSD severity, based on re-rating 112 recordings across different assessment timepoints).

**Figure 2:**
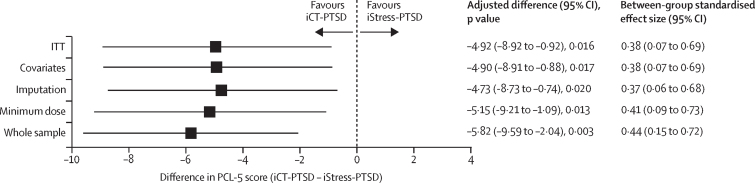
Adjusted mean differences between iCT-PTSD and iCT-Stress for the primary and sensitivity analyses of PTSD symptoms assessed at 13 weeks PTSD symptoms were assessed with the self-reported PCL-5. ITT analysis includes all available data for immediate allocation, controlled for baseline PCL-5, months since the trauma (log), and site. Covariates analysis is the same as ITT but with a variable predicting missing data as an additional covariate (SAPAS^33^). Imputation analysis is multiple imputation of the missing nine datapoints. Minimum dose analysis excludes participants who did not have a minimum dose of therapy (ie, completed core procedures relating to psychoeducation, activities in everyday life, and at least one of the core technique addressing trauma memories in iCT or one core stress-management technique in iStress; appendix p 23). Whole sample analysis includes allocations after the waiting list period. iCT-PTSD=internet-delivered cognitive therapy for PTSD. iStress-PTSD=internet-based stress management therapy for PTSD. ITT=intention-to-treat. PCL-5= PTSD Checklist for DSM-5. PTSD=post–traumatic stress disorder. SAPAS=Structured Assessment of Personality Abbreviated Scale

The primary intention-to-treat analysis, which included the 185 participants immediately allocated to treatment, showed that iCT-PTSD was superior to iStress-PTSD with an adjusted difference on the PCL-5 of –4·92 (95% CI –8·92 to –0·92; *d*=0·38 [0·07 to 0·69]; figure 2; appendix p 15). Nine (4%) of all 212 participants allocated to treatment had missing data for the primary outcome at 13 weeks, and only one variable (higher scores on the Standardised Assessment of Personality Abbreviated Scale)^33^ predicted missingness and was included in the statistical model for the first sensitivity analysis. This analysis and all other sensitivity analyses support that iCT-PTSD was superior to iStress-PTSD (figure 2).

Both treatments were superior to the waiting list control on the PCL-5 at 13 weeks; the adjusted difference compared with the waiting list were 20·98 points (95% CI 15·50–26·45) for iCT-PTSD (*d*=1·67 [95% CI 1·23–2·10]) and 16·19 points (10·74–21·63) for iStress-PTSD (*d*=1·29 [0·85–1·72]; appendix pp 15–19). The mean percentage change in PCL-5 scores at 13 weeks was 68% (SD 27) for iCT-PTSD, 57% (SD 31) for iStress-PTSD, and 21% (SD 27) for the waiting list group. Both treatments were superior to the waiting list control for secondary PTSD measures and other secondary outcomes (appendix pp 15–19).

Across outcomes, iCT-PTSD and iStress-PTSD led to similar improvements across measures up to the 6-week assessment (table 2), but at 13 weeks, iCT-PTSD was superior to iStress-PTSD in symptoms of PTSD, depression, anxiety, and disability. At 13 weeks, the standardised within-group effect size was 2·22 (95% CI 2·02–2·42) for iCT-PTSD and 1·85 (1·66–2·05) for iStress-PTSD. By 26 weeks, the iCT-PTSD group also showed superior outcomes in quality of life and wellbeing, but there were no differences between iCT-PTSD and iStress-PTSD in improvement in sleep quality at any timepoint (table 2).

**Table 2:** Comparisons between iCT-PTSD and iStress-PTSD on the primary and secondary outcome measures (intention-to-treat analysis)

	**Unadjusted mean (SD); N**		**Adjusted difference (95% CI)**	**p value**	**Standardised between-group effect size (95% CI)**	**Standardised within-group effect size (95% CI)**	
	iCT–PTSD	iStress–PTSD				iCT–PTSD	iStress–PTSD
**PTSD severity (PCL-5)**							
Baseline	45·03 (13·74); 107	46·83 (12·73); 105	..	..	..	..	..
6 weeks	26·77 (16·51); 98	26·83 (15·99); 96	0·90 (−2·91 to 4·71)	0·642	0·07 (−0·22 to 0·36)	1·35 (1·15 to 1·55)	1·50 (1·29 to 1·70)
13 weeks	15·41 (14·02); 100	22·67 (17·39); 101	−5·82 (−9·59 to −2·04)	0·0027	0·44 (0·15 to 0·72)	2·22 (2·02 to 2·42)	1·85 (1·66 to 2·05)
26 weeks	11·45 (11·77); 93	18·76 (17·04); 94	−6·82 (−10·67 to −2·98)	0·0006	0·52 (0·22 to 0·81)	2·49 (2·29 to 2·69)	2·06 (1·86 to 2·26)
**PTSD severity (IES-R)**							
Baseline	53·08 (14·74); 107	53·32 (14·79); 105	..	..	..	..	..
6 weeks	28·98 (19·09); 96	30·88 (18·25); 96	−0·53 (−5·05 to 3·98)	0·816	0·04 (−0·27 to 0·34)	1·58 (1·37 to 1·79)	1·52 (1·31 to 1·74)
13 weeks	15·74 (15·49); 98	25·66 (19·97); 100	−8·30 (−12·78 to −3·81)	0·0003	0·56 (0·26 to 0·87)	2·48 (2·27 to 2·69)	1·90 (1·69 to 2·11)
26 weeks	12·71 (14·19); 93	20·13 (18·24); 94	−6·88 (−11·42 to −2·33)	0·0032	0·47 (0·16 to 0·78)	2·69 (2·48 to 2·91)	2·21 (2·00 to 2·43)
**PTSD severity, assessor (CAPS-5)**							
Baseline	40·20 (9·98); 107	40·51 (9·50); 105	..	..	..	..	..
13 weeks	14·79 (12·33); 92	18·61 (14·33); 93	−3·43 (−6·85 to −0·01)	0·049	0·35 (<0·01 to 0·70)	2·59 (2·34 to 2·84)	2·22 (1·97 to 2·47)
26 weeks	12·51 (12·32); 91	17·89 (14·38); 88	−5·32 (−8·77 to −1·87)	0·0027	0·55 (0·19 to 0·90)	2·84 (2·59 to 3·09)	2·29 (2·04 to 2·55)
**Depression (PHQ-9)**							
Baseline	12·93 (5·89); 107	13·61 (6·12); 105	..	..	..	..	..
6 weeks	9·55 (6·44); 98	9·20 (5·88); 95	0·46 (−1·00 to 1·91)	0·536	0·08 (−0·17 to 0·32)	0·55 (0·37 to 0·73)	0·70 (0·52 to 0·88)
13 weeks	6·39 (5·76); 100	8·50 (6·58); 101	−1·77 (−3·21 to −0·33)	0·016	0·30 (0·05 to 0·53)	1·09 (0·91 to 1·27)	0·87 (0·70 to 1·05)
26 weeks	4·68 (5·09); 93	6·52 (5·86); 94	−1·88 (−3·35 to 0·41)	0·012	0·31 (0·07 to 0·56)	1·35 (1·17 to 1·53)	1·12 (0·93 to 1·30)
**Anxiety (GAD-7)**							
Baseline	12·36 (5·23); 107	13·15 (5·40); 105	..	..	..	..	..
6 weeks	8·66 (5·65); 96	8·54 (5·42); 95	0·46 (−0·93 to 1·84)	0·516	0·09 (−0·17 to 0·35)	0·67 (0·47 to 0·87)	0·84 (0·64 to 1·04)
13 weeks	5·52 (4·97); 99	7·64 (6·22); 101	−1·63 (−3·00 to −0·27)	0·019	0·31 (0·05 to 0·56)	1·28 (1·09 to 1·48)	1·07 (0·88 to 1·26)
26 weeks	4·30 (4·73); 93	6·06 (5·86); 94	−1·73 (−3·12 to −0·33)	0·015	0·32 (0·06 to 0·59)	1·50 (1·30 to 1·69)	1·27 (1·08 to 1·47)
**Disability (WSAS)**							
Baseline	18·91 (8·61); 107	19·14 (8·69); 105	..	..	..	..	..
6 weeks	13·66 (9·04); 96	15·45 (9·77); 95	−1·27 (−3·45 to 0·91)	0·253	0·15 (−0·11 to 0·40)	0·55 (0·36 to 0·74)	0·40 (0·21 to 0·59)
13 weeks	9·55 (8·81); 98	12·97 (10·34); 101	−2·66 (−4·81 to −0·51)	0·016	0·31 (0·06 to 0·56)	1·06 (0·87 to 1·24)	0·74 (0·55 to 0·92)
26 weeks	7·98 (8·97); 93	9·43 (9·26); 94	−1·61 (−3·80 to 0·59)	0·151	0·19 (−0·07 to 0·44)	1·26 (1·07 to 1·45)	1·08 (0·88 to 1·27)
**Wellbeing (WHO-5)**							
Baseline	7·97 (4·21); 107	7·16 (4·60); 105	..	..	..	..	..
6 weeks	11·50 (5·30); 74	10·26 (4·77); 78	0·75 (−0·74 to 2·25)	0·320	0·17 (−0·17 to 0·51)	0·74 (0·50 to 0·98)	0·64 (0·40 to 0·88)
13 weeks	12·95 (5·79); 92	11·45 (5·86); 92	0·72 (−0·69 to 2·12)	0·314	0·16 (−0·16 to 0·48)	1·07 (0·85 to 1·30)	0·97 (0·74 to 1·19)
26 weeks	14·60 (5·57); 86	12·31 (5·77); 87	2·15 (0·72 to 3·58)	0·003	0·49 (0·16 to 0·81)	1·53 (1·30 to 1·76)	1·13 (0·90 to 1·36)
**General quality of life (Q-LES-Q)**							
Baseline	42·26 (9·88); 107	41·50 (9·52); 105	..	..	..	..	..
6 weeks	48·68 (11·77); 74	47·32 (10·24); 78	0·78 (−2·12 to 3·68)	0·598	0·08 (−0·22 to 0·38)	0·63 (0·42 to 0·84)	0·58 (0·38 to 0·79)
13 weeks	52·02 (11·99); 92	49·97 (11·84); 92	1·22 (−1·51 to 3·95)	0·378	0·13 (−0·16 to 0·41)	0·98 (0·79 to 1·18)	0·89 (0·69 to 1·08)
26 weeks	54·23 (11·03); 86	50·39 (11·40); 87	3·77 (0·99 to 6·55)	0·0082	0·39 (0·10 to 0·68)	1·26 (1·06 to 1·46)	0·91 (0·71 to 1·11)
**Health-related quality of life (5-level EQ-5D version)**							
Baseline	0·61 (0·25); 106	0·60 (0·25); 105	..	..	..	..	..
13 weeks	0·72 (0·24); 91	0·69 (0·24); 92	0·02 (−0·04 to 0·07)	0·457	0·08 (−0·16 to 0·30)	0·42 (0·24 to 0·60)	0·35 (0·20 to 0·52)
26 weeks	0·77 (0·23); 86	0·70 (0·28); 86	0·07 (0·01 to 0·13)	0·023	0·27 (0·04 to 0·52)	0·66 (0·48 to 0·84)	0·40 (0·24 to 0·56)
**Sleep Disturbance (ISI)**							
Baseline	15·12 (7·14); 107	16·33 (6·24); 105	..	..	..	..	..
6 weeks	10·85 (6·94); 93	11·99 (6·97); 94	0·12 (−1·49 to 1·73)	0·887	0·02 (−0·22 to 0·26)	0·56 (0·38 to 0·74)	0·64 (0·46 to 0·82)
13 weeks	8·90 (7·14); 96	9·66 (7·11); 99	0·39 (−1·20 to 1·98)	0·626	0·06 (−0·18 to 0·29)	0·86 (0·69 to 1·04)	0·99 (0·82 to 1·17)
26 weeks	8·02 (6·43); 93	8·68 (6·16); 94	−0·12 (−1·72 to 1·49)	0·888	0·02 (−0·22 to 0·26)	1·05 (0·87 to 1·22)	1·10 (0·92 to 1·28)

The table shows the observed unadjusted means for each group. All statistics are based on intent-to-treat analyses using linear mixed-effect models. Within-group effect sizes represent change from baseline in each group. Adjusted mean differences based on linear mixed-effects models adjusted for baseline scores and stratification variables. Standardised effect sizes calculated using the baseline SD of whole sample. CAPS-5=Clinician-Administered PTSD Scale for DSM-5. GAD-7=General Anxiety Disorder-7. iCT-PTSD=internet-delivered cognitive therapy for PTSD. iStress-PTSD=internet-based stress management therapy for PTSD. IES-R=Impact of Event Scale-Revised. ISI=Insomnia Severity Index. PCL-5=PTSD Checklist for DSM-5. PHQ-9=9-question Patient Health Questionnaire. PTSD=post–traumatic stress disorder. Q-LES-Q=Quality of Life Enjoyment and Satisfaction Questionnaire. WHO-5=World Health Organization Five Well-Being Index. WSAS=Work and Social Adjustment Scale.

The IAPT Patient Experience Questionnaire^30^ at 13 weeks showed high satisfaction for both treatments, with iCT-PTSD scoring significantly higher (p=0·0061) than iStress-PTSD (appendix p 11). In their open responses, participants reported that they greatly valued their therapist's support through calls and messages, and several participants commented that they preferred doing therapy in their own time; further themes are in the appendix (p 32).

During follow-up (39 weeks and 65 weeks), iCT-PTSD remained superior to iStress-PTSD across all outcome measures, and there continued to be no difference in sleep quality (appendix pp 13–14). Large to very large standardised within-group effect sizes show that the benefits of treatment were maintained in both groups. The difference between the treatments in general quality of life (Q-LES-Q^27^) increased from 0·13 at 13 weeks and 0·39 at 26 weeks to 0·48 at 65 weeks (table 2; appendix p 14).

A priori established dichotomous outcome criteria were based on those commonly reported in the literature and national statistics for IAPT services. At 13 weeks, the intention-to-treat rate for clinically significant change on the PCL-5 (ie, a decrease of ≥10 points and post-treatment scores >2 SD below the group baseline score)^37^ was greater for iCT-PTSD (76 [71%] of 107 participants) than for iStress-PTSD (52 [50%] of 105 participants; table 3). We also found higher rates in the iCT-PTSD group for no longer meeting PTSD diagnosis criteria according to DSM-5 (assessed by CAPS-5 score) and for IAPT recovery,^30^ which requires both PTSD and depression symptoms to move into the non-clinical range (table 3). Some participants in the waiting list group also showed clinically significant change (appendix p 20), and three (10%) of 31 participants in the waiting list group showed symptom deterioration on the main outcome measure (defined as an increase of at least 5 points on the PCL-5; appendix p 20) compared with none in the iCT-PTSD group and one (1%) participant in the iStress-PTSD group. There were no serious treatment-related adverse events in any of the groups (appendix p 24).

**Table 3:** Dichotomous criteria for improvement and deterioration: outcomes for iCT-PTSD and iStress-PTSD (all allocations)

		**iCT–PTSD (n=107)**	**iStress-PTSD (n=105)**
**Self-reported PTSD symptoms**			
ITT clinically significant change in PCL-5*			
	13 weeks	76/107 (71%)	52/105 (50%)
	26 weeks	80/107 (75%)	59/105 (56%)
	39 weeks	79/107 (74%)	62/105 (59%)
	65 weeks	77/107 (72%)	59/105 (56%)
Asymptomatic on PCL-5†			
	13 weeks	46/100 (46%)	29/101 (29%)
	26 weeks	52/93 (56%)	39/94 (42%)
	39 weeks	53/92 (58%)	38/93 (41%)
	65 weeks	55/95 (58%)	40/97 (41%)
Worsening in PTSD symptoms on the PCL-5 compared with baseline‡			
	13 weeks	0	2/101 (2%)
	26 weeks	0	4/94 (4%)
	39 weeks	0	5/93 (5%)
	65 weeks	2/95 (2%)	4/97 (4%)
No longer met criteria for PTSD diagnosis (CAPS-5)§			
	13 weeks	77/92 (84%)	64/93 (69%)
	26 weeks	77/91 (85%)	64/88 (73%)
	39 weeks	75/85 (88%)	59/86 (69%)
	65 weeks	70/83 (84%)	61/84 (73%)
Asymptomatic on CAPS-5¶			
	13 weeks	42/92 (46%)	31/93 (33%)
	26 weeks	51/91 (56%)	34/88 (39%)
	39 weeks	44/85 (52%)	33/86 (38%)
	65 weeks	46/83 (55%)	38/84 (45%)
Worsening in CAPS-5 scores compared with baseline‖			
	13 weeks	0	4/93 (4%)
	26 weeks	1/91 (1% )	3/88 (2%)
	39 weeks	1/85 (1%)	5/86 (6%)
	65 weeks	2/83 (2%)	5/84 (6%)
**IAPT criteria (PTSD and depression symptoms)**			
IAPT recovery**			
	Last assessment at end of therapy (ie, at 26 weeks or earlier depending on when participants finished)	81/105 (77%)	64/102 (63%)
IAPT reliable improvement††			
	Last assessment at end of therapy (ie, at 26 weeks or earlier depending on when participants finished)	98/104 (94%)	87/104 (84%)
IAPT reliable deterioration‡‡			
	Last assessment at end of therapy (ie, at 26 weeks or earlier depending on when participants finished)	0	4/104 (4%))

CAPS-5=Clinician-Administered PTSD Scale for DSM-5. IAPT=Improving Access to Psychological Therapies. iCT-PTSD=internet-delivered cognitive therapy for PTSD. iStress-PTSD=internet-based stress management therapy for PTSD. ITT=intention-to-treat. PCL-5=PTSD Checklist for DSM-5. PHQ-9=9-item Patient Health Questionnaire. PTSD=post–traumatic stress disorder.

* Defined as a reliable change of ≥10 points on the PCL-5 and a score lower than 2 SDs below the mean of treatment sample at baseline.^37^ For the ITT analysis, missing data were scored as no clinically significant change unless there was clear evidence from CAPS-5 or scores in online programme.

† Defined as scoring ≤10 points on the PCL-5 and calculated for participants with available data.

‡ Defined as worsening by ≥5 points on the PCL-5 and calculated for participants with available data.

§ Defined as meeting all DSM-5 PTSD criteria with a threshold of 2 points for symptoms and interference ratings on CAPS-5 items, and calculated for participants with available data.

¶ Defined as having ≤10 points on CAPS-5 and calculated for participants with available data.

‖ Defined as worsening by ≥5 points on the CAPS-5 and calculated for participants with available data.

** Scoring according to the IAPT manual^30^ as PCL-5 <32 points and PHQ-9 <10 points; missing data scored as no recovery; denominator excludes patients with subthreshold PCL or PHQ-9 scores at baseline.

†† Scoring according to the IAPT manual^30^ as PCL-5 change of ≥10 points or PHQ-9 change of ≥6 points, and no reliable deterioration in either measure; for patients with at least two datapoints; four patients had missing data (eg, did not start treatment or missing questionnaire).

‡‡ Scoring according to the IAPT manual^30^ as PCL-5 change of ≤–10 points or PHQ-9 change of ≤–6 points, and no reliable improvement in either measure; for patients with at least two datapoints; four patients had missing data (eg, did not start treatment or missing questionnaire).

Moderation analyses showed that for participants with high dissociation and those meeting self-reported criteria for complex PTSD,^32^ the advantage of iCT-PTSD over iStress-PTSD was greater than for those with lower scores (appendix p 24). Demographics, comorbidity, and trauma history did not moderate the outcome (appendix p 25). Mediation analyses (appendix pp 27–30) showed that both treatments worked by changing negative meanings of the trauma, and by reducing unhelpful coping and flashback memories, in line with theoretical predictions.^19^ An exploratory analysis (appendix p 31) suggested that participants in the iStress-PTSD condition who chose to work on trauma-related exposure hierarchies had better outcomes than those who worked on other hierarchies.

## Discussion

To our knowledge, this is the first study to compare the efficacy of a trauma-focused and non-trauma-focused therapist-assisted online cognitive behavioural treatment for people with PTSD as diagnosed using DSM-5. iCT-PTSD, a novel treatment that incorporates all procedures of NICE recommended CT-PTSD,^4^ was superior to iStress-PTSD, a comprehensive CBT stress-management programme focused on teaching a wide range of strategies to manage stress and PTSD symptoms.^5^ Since iCT-PTSD and iStress-PTSD were rated as similarly credible and had similarly high therapeutic alliance, these findings suggest that a therapist-assisted, internet-delivered treatment for PTSD has specific therapeutic effects and leads to greater satisfaction with treatment.

The advantage of iCT-PTSD over iStress-PTSD was maintained during follow-up. iCT-PTSD led to substantial and lasting reductions in PTSD symptoms and a wide range of other outcomes, including quality of life. For participants in the iCT-PTSD group, the standardised within-group effect sizes in reduction of PTSD symptoms of 2·22 at 13 weeks to 2·49 at 26 weeks and the proportion of participants who no longer met criteria for a DSM-5 PTSD diagnosis (84% of participants who completed the 13-week assessment and 72% of participants who were randomly assigned) are in line with those observed in RCTs for face-to-face CT-PTSD.^4^, ^11^ This finding suggests that online treatment delivery did not compromise outcome. However, iCT-PTSD requires on average less than half of the usual therapist time, saving therapist costs per patient treated.

Our results parallel the findings of meta-analyses for face-to-face psychological treatments for PTSD^3^ and suggest that trauma-focused treatments should be the treatments of choice for therapist-assisted internet-based therapies. The specific procedures of iCT-PTSD that help patients update threatening meanings of their traumas and discriminate triggers had additional therapeutic effects beyond those of good generic coping-focused CBT. The exploratory analysis of iStress-PTSD is also consistent with the conclusion that a trauma focus enhances outcomes: participants who chose to expose themselves to reminders of the trauma had better outcomes than participants who worked on other hierarchies of avoided situations.

Both treatments were efficacious and superior to waiting for treatment with usual NHS care. The comprehensiveness of the treatments, each implementing the same treatment procedures as for face-to-face therapy, and the quality and frequency of support provided by therapists is a probable factor in these results. Participants reported that they greatly valued their therapist's support through calls and messages, and the treatments were clearly acceptable to patients since satisfaction ratings were high and dropout rates were below 10%. This rate compares favourably with the mean dropout rate of 23% reported for therapist-assisted iCBT^6^ and a mean of 31% for internet-based treatments for psychological disorders in general.^38^

Although some clinicians might be concerned that remote delivery of a trauma-focused treatment could increase the probability of symptom deterioration, our findings suggest otherwise, as PTSD symptom deterioration occurred in about 10% of participants randomly assigned to waiting list with usual care and in none of the participants assigned to iCT-PTSD, replicating the findings for face-to-face CT-PTSD.^11^

The moderation analyses showed that the additional benefits for iCT-PTSD over iStress-PTSD were especially large for participants with high dissociation and self-reported complex PTSD symptoms, which contradicts any expectation that trauma-focused treatments might be less tolerable or less beneficial for people with severe presentations of PTSD.

Four participants in the waiting list group no longer met full criteria for PTSD at the end of the waiting list period. In line with epidemiological data on natural recovery over time,^13^ three of these participants had recent traumas (ie, <6 months ago) and none were taking psychotropic medication. iStress-PTSD and iCT-PTSD shared common non-specific elements of good psychotherapy such as building a therapeutic relationship and support (highlighted as important by participants; appendix p 32) and common elements of good CBT such as psychoeducation, collaborative work towards the client's goals, and learning new cognitive and behavioural skills to deal with symptoms and resulting problems. The specific effects of working on trauma memories, triggers of those memories, and personal meanings of the trauma in iCT-PTSD were seen once participants had made progress with the memory-focused work, which is similar to findings in other studies comparing specific with non-specific CBT in other disorders.^39^ One could ask whether the small to medium effects matter. The increasing differences in quality of life between the treatments during follow-up suggest that they do. However, for subgroups such as people with mild symptoms treated soon after a trauma, detailed work on trauma memories might not be needed.

The mediation analysis results support the importance of changing the psychological processes in the theoretical model that underpins CT-PTSD.^19^ By contrast, self-efficacy did not mediate differences in outcome between the treatments (appendix p 29). This finding supports the specificity of the cognitive processes that drive PTSD symptoms and recovery.

Therapist-assisted online treatments provide convenience for patients because they can work on the treatment in a place and at a time that suits them. Several participants commented that they preferred doing therapy in their own time (appendix p 33). Another possible advantage of online treatment programmes is that treatment fidelity might be more consistent across therapists than for face-to-face therapy when delivered in routine clinical practice, because the content of the internet treatments is mainly delivered through online modules, which ensure all relevant treatment procedures are covered. This advantage has implications for ensuring consistency in content and quality of treatment delivery across a large workforce with varying amounts of training in the treatment of PTSD such as IAPT. The mean IAPT recovery rate for PTSD in services across England was 40% in 2020–21 and 41% in 2021–22, compared with 77% for iCT-PTSD in this study and similar outcomes achieved in a recent unpublished implementation study with IAPT therapists.^40^

This study had various limitations. Although iStress-PTSD was chosen to represent a non-trauma-focused treatment, it contains some trauma-related content, such as normalising the effects of traumatic events as stress reactions and the application of coping strategies to intrusive trauma memories. Some participants also used coping skills such as challenging their thoughts and exposure to trauma-related thoughts and situations, which contributed to the efficacy of iStress-PTSD and ensured the credibility of the treatment, but might have underestimated the difference between treatments. The analysis of treatment effects and their maintenance focused on group means, and a small group of individual participants showed different patterns such as deterioration or continued improvement during follow-up. Negative life events and the COVID-19 pandemic affected some participants’ treatment and assessments. In clinical practice, communication by phone calls and messages might not suit everyone, and therapists could consider offering a choice between video and phone calls, or blend iCT-PTSD modules with some face-to-face sessions.

Overall, our results suggest that iCT-PTSD is a viable and promising alternative to face-to-face therapy for PTSD and could contribute to increasing patient choice, among other options such as other trauma-focused iCBT programmes.^8^, ^9^ iStress-PTSD could be of interest to patients who do not wish to talk about their trauma.

## Data sharing

Trial materials can be obtained from the AE. Given the highly personal nature of the study data, participants were asked for optional consent to sharing their anonymised data with other researchers. Many, but not all, participants consented, and their deidentified and anonymised data will be available from the AE upon reasonable request, subject to submission and approval of a research proposal and review and contract with the University of Oxford (Oxford, UK), following the publication of all results from this study.

For the **trial protocol** see https://trialsjournal.biomedcentral.com/articles/10.1186/s13063-020-4176-8

## Declaration of interests

## References

[1] National Institute for Health and Care Excellence (2018). Post-traumatic stress disorder. https://www.nice.org.uk/guidance/ng116.

[2] International Society of Traumatic Stress Studies (2019). Posttraumatic stress disorder prevention and treatment guidelines. http://www.istss.org/getattachment/Treating-Trauma/New-ISTSS-Prevention-and-Treatment-Guidelines/ISTSS_PreventionTreatmentGuidelines_FNL.pdf.aspx.

[3] Bisson JI, Roberts NP, Andrew M, Cooper R, Lewis C (2013). Psychological therapies for chronic post-traumatic stress disorder (PTSD) in adults. Cochrane Database Syst Rev.

[4] Ehlers A, Clark DM, Hackmann A, McManus F, Fennell M (2005). Cognitive therapy for PTSD: development and evaluation. Behav Res Ther.

[5] Persson Asplund R, Asplund S, von Buxhoeveden H (2023). Work-focused versus generic internet-based interventions for employees with stress-related disorders: randomized controlled trial. J Med Internet Res.

[6] Simon N, Robertson L, Lewis C (2021). Internet-based cognitive and behavioural therapies for post-traumatic stress disorder (PTSD) in adults. Cochrane Database Syst Rev.

[7] Lewis CE, Farewell D, Groves V (2017). Internet-based guided self-help for posttraumatic stress disorder (PTSD): randomized controlled trial. Depress Anxiety.

[8] Knaevelsrud C, Brand J, Lange A, Ruwaard J, Wagner B (2015). Web-based psychotherapy for posttraumatic stress disorder in war-traumatized Arab patients: randomized controlled trial. J Med Internet Res.

[9] Bisson JI, Ariti C, Cullen K (2022). Guided, internet based, cognitive behavioural therapy for post-traumatic stress disorder: pragmatic, multicentre, randomised controlled non-inferiority trial (RAPID). BMJ.

[10] Wild J, Warnock-Parkes E, Grey N (2016). Internet-delivered cognitive therapy for PTSD: a development pilot series. Eur J Psychotraumatol.

[11] Ehlers A, Hackmann A, Grey N (2014). A randomized controlled trial of 7-day intensive and standard weekly cognitive therapy for PTSD and emotion-focused supportive therapy. Am J Psychiatry.

[12] Persson Asplund R, Dagöö J, Fjellström I (2018). Internet-based stress management for distressed managers: results from a randomised controlled trial. Occup Environ Med.

[13] Kessler RC, Sonnega A, Bromet E, Hughes M, Nelson CB (1995). Posttraumatic stress disorder in the National Comorbidity Survey. Arch Gen Psychiatry.

[14] Ehlers A, Wild J, Warnock-Parkes E (2020). A randomised controlled trial of therapist-assisted online psychological therapies for posttraumatic stress disorder (STOP-PTSD): trial protocol. Trials.

[15] First MB, Williams JBW, Karg RS, Spitzer RL (2017).

[16] First MB, Williams JBW, Benjamin LS, Spitzer RL (2017).

[17] Borkovec TD, Nau SD (1972). Credibility of analogue therapy rationales. J Behav Ther Exp Psychiatry.

[18] Horvath AO, Greenberg LS (1989). Development and validation of the Working Alliance Inventory. J Couns Psychol.

[19] Ehlers A, Clark DM (2000). A cognitive model of posttraumatic stress disorder. Behav Res Ther.

[20] National Center for PTSD Using the PTSD checklist for DSM-5 (PCL-5). https://www.ptsd.va.gov/professional/assessment/documents/using-PCL5.pdf.

[21] Weiss DS, Marmar CR, Wilson J, Keane TM (1996). Assessing psychological trauma and PTSD.

[22] Weathers FW, Blake DD, Schnurr PP, Kaloupek DG, Marx BP, Keane TM (2013). The Clinician-Administered PTSD Scale for DSM-5 (CAPS-5). http://www.ptsd.va.gov.

[23] Kroenke K, Spitzer RL, Williams JBW (2001). The PHQ-9: validity of a brief depression severity measure. J Gen Intern Med.

[24] Spitzer RL, Kroenke K, Williams JB, Löwe B (2006). A brief measure for assessing generalized anxiety disorder: the GAD-7. Arch Intern Med.

[25] Mundt JC, Marks IM, Shear MK, Greist JH (2002). The Work and Social Adjustment Scale: a simple measure of impairment in functioning. Br J Psychiatry.

[26] Topp CW, Østergaard SD, Søndergaard S, Bech P (2015). The WHO-5 Well-Being Index: a systematic review of the literature. Psychother Psychosom.

[27] Rapaport MH, Clary C, Fayyad R, Endicott J (2005). Quality-of-life impairment in depressive and anxiety disorders. Am J Psychiatry.

[28] Morin CM, Belleville G, Bélanger L, Ivers H (2011). The Insomnia Severity Index: psychometric indicators to detect insomnia cases and evaluate treatment response. Sleep.

[29] Herdman M, Gudex C, Lloyd A (2011). Development and preliminary testing of the new five-level version of EQ-5D (EQ-5D-5L). Qual Life Res.

[30] The Improving Access to Psychological Therapies Manual (2018). Appendices and helpful resources. https://www.england.nhs.uk/wp-content/uploads/2018/06/iapt-manual-resources-v2.pdf.

[31] Weathers FW, Blake DD, Schnurr PP, Kaloupek DG, Marx BP, Keane TM (2013). The Life Events Checklist for DSM-5 (LEC-5). http://www.ptsd.va.gov.

[32] Hyland P, Shevlin M, Brewin CR (2017). Validation of post-traumatic stress disorder (PTSD) and complex PTSD using the International Trauma Questionnaire. Acta Psychiatr Scand.

[33] Moran P, Leese M, Lee T, Walters P, Thornicroft G, Mann A (2003). Standardised Assessment of Personality—Abbreviated Scale (SAPAS): preliminary validation of a brief screen for personality disorder. Br J Psychiatry.

[34] Babor TF, Higgins-Biddle JC, Saunders JB, Monteiro MG (2001).

[35] National Institute for Health and Care Excellence (2005).

[36] Murray J, Ehlers A, Mayou RA (2002). Dissociation and post-traumatic stress disorder: two prospective studies of road traffic accident survivors. Br J Psychiatry.

[37] Jacobson NS, Truax P (1991). Clinical significance: a statistical approach to defining meaningful change in psychotherapy research. J Consult Clin Psychol.

[38] Melville KM, Casey LM, Kavanagh DJ (2010). Dropout from Internet-based treatment for psychological disorders. Br J Clin Psychol.

[39] Ljótsson B, Hesser H, Andersson E (2013). Mechanisms of change in an exposure-based treatment for irritable bowel syndrome. J Consult Clin Psychol.

[40] NHS Digital Psychological therapies, annual reports on the use of IAPT services. https://digital.nhs.uk/data-and-information/publications/statistical/psychological-therapies-annual-reports-on-the-use-of-iapt-services.

