# Supplementary material for: Therapist-assisted online psychological therapies differing in trauma focus for post-traumatic stress disorder (STOP-PTSD): a UK-based, single-blind, randomised controlled trial
Source: Lancet Psychiatry. 2023 Aug;10(8):608–22. doi: 10.1016/S2215-0366(23)00181-5 (PMC10789612; doi:10.1016/S2215-0366(23)00181-5)
Supplement: Supplementary appendix [file mmc1.pdf]

THE LANCET
Psychiatry

Supplementary appendix

This appendix formed part of the original submission and has been peer reviewed. We post it as supplied by the authors.

Supplement to: Ehlers A, Wild J, Warnock-Parkes E, et al. Therapist-assisted online psychological therapies differing in trauma focus for post-traumatic stress disorder (STOP-PTSD): a UK-based, single-blind, randomised controlled trial. *Lancet Psychiatry* 2023; **10:** 608–22.

Online Appendix: thelancetpsych-D-23-00191

A randomised controlled trial of therapist-assisted online psychological therapies for post-traumatic stress disorder differing in trauma-focus (STOP-PTSD)
(Ehlers et al., 2023)

Table of Contents

Supplement	Page
1: STOP-PTSD inclusion and exclusion criteria, referrals	2
**2:** Detailed CONSORT Flow-chart	3
**3:** Further descriptions of the treatments	4
**4:** Reasons given for dropping out of treatment or follow-up (all allocated to treatment)	9
**5:** Treatment compliance, acceptability and satisfaction, working alliance, and time commitment	10
**6**: Maintenance of Treatment Effects: Comparisons between iCT-PTSD and iStress-PTSD on the primary and secondary outcome measures (all allocated to treatment)	13
**7.** Comparisons between each of the treatments and waitlist with usual NHS care on the primary and secondary outcome measures at 13 weeks	15
**8.** Dichotomous criteria for improvement and deterioration for comparisons of the treatments with wait with usual NHS care	20
**9.** CACE analysis	23
**10.** Adverse events and adversity during treatment	24
**11:** Moderation analyses	25
**12:** Mediation analyses	27
**13:** Exploratory analysis: Trauma-focused use of exposure in iStress-PTSD	31
**14:** Examples of themes from interviews about patient experience	32
**15:** Primary outcome PCL-5 by gender	34
**16:** Changes in medication and other therapies accessed during treatment	35
**17:** Statistical analysis plan	37

Supplement 1 STOP-PTSD Inclusion and Exclusion Criteria

Inclusion Criteria

Participants must meet the following criteria:

1. Aged 18 and above.
2. Willing and able to provide informed consent.
3. Meets diagnostic criteria for PTSD as determined by the Structured Clinical Interview for DSM-5 [28].
4. Their current reexperiencing symptoms are linked to one or two discrete traumatic events that they experienced in adulthood or adolescence, or several traumatic episodes during a longer period of high threat (e.g., domestic abuse, war zone experiences).
5. PTSD is the main psychological problem needing treatment.
6. Able to read and write in English.
7. Access to the internet.
8. Willing to be randomly allocated to one of the psychological treatments or wait.
9. If taking psychotropic medication, the dose must be stable for at least 1 month before randomisation.
10. If currently receiving psychological therapy for PTSD, this treatment must have ended before randomisation

Exclusion Criteria

A person is not eligible if any of the following apply (assessed by clinician in the initial clinical assessment).

1. History of psychosis.
2. Current substance dependence.
3. Current borderline personality disorder.
4. Acute serious suicide risk.

Participants were mainly recruited from *Improving Access to Psychological Therapies* (IAPT)[1] services (n=212) in rural and urban areas (Buckinghamshire, Berkshire, Croydon, Lambeth, Lewisham, Oxfordshire, Southwark, Brighton & Hove, and East Sussex). Participants in the same areas could also self-refer (n=35) in response to information listed on study and trial registration websites (ISRCTN; UK Clinical Trials Gateway), based on findings that self-referrals are more representative of the ethnic mix in the community, and have the same severity and longer duration of problems than GP referrals (Clark et al., 2009). IAPT services allow self-referrals but people in the community are not always aware of this.

Reference

Clark DM, Layard R, Smithies R, Richards DA, Suckling R, Wright B. Improving access to psychological therapy: Initial evaluation of two UK demonstration sites. Behav Res Ther 2009; 47(11):910-20. doi: 10.1016/j.brat.2009.07.010

[1] In 2023, NHS England renamed IAPT as *NHS Talking Therapies for Anxiety and Depression.*

Supplement 2: Detailed CONSORT Flow chart

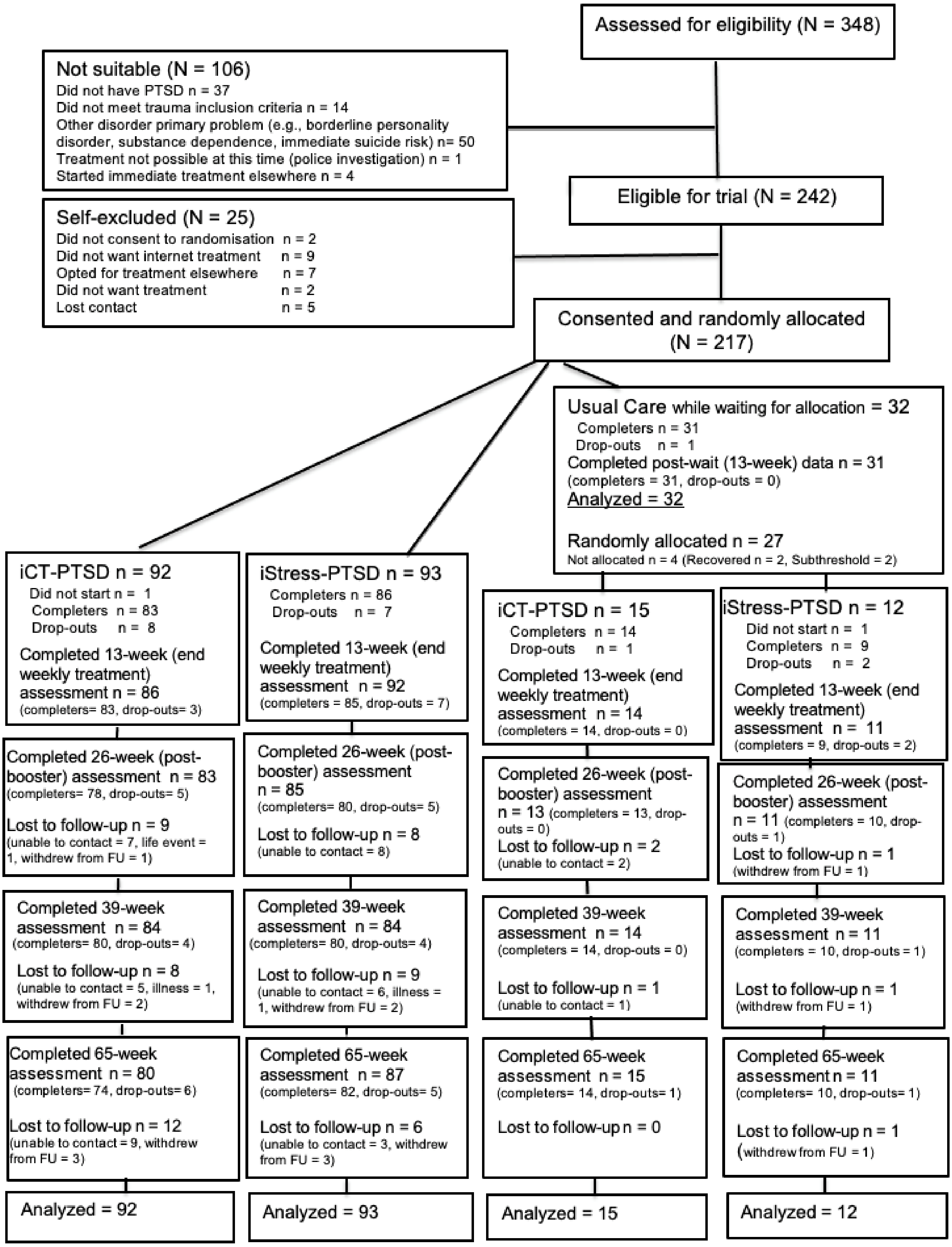

Supplement 3: Description of interventions

Both internet-delivered treatments have the same user interface and design, and can be accessed by PC, tablets or smart phones. Participants who did not have a PC or tablet could borrow a tablet for the duration of treatment. The modules for both interventions were identical in design and shared the same multimedia features to facilitate engagement and accessibility. The modules and design were developed with extensive input from service users. Both interventions are delivered via online modules that are identical in design and share the same multimedia features to facilitate engagement and accessibility (text with information and patient examples, videos of a therapist talking or whiteboard videos for key information, videos of patient testimonies, questions and text boxes for the patient to complete, audio-recordings for the patient to listen to, graphs and pictures).

iCT-PTSD and iStress-PTSD were delivered online via a series of therapy modules with therapist support by messages within the programme, SMS and short weekly phone calls (designed to last on average 20 min) over the first 12 weeks (weekly treatment phase) and 3 phone calls over the next 3 months (booster phase). The therapist released the modules gradually, 2 to 3 modules per week. Therapists could, with the participant's knowledge, read the information that they provided in the modules and write notes for the participants directly into the modules.

Participants retained access to the programme for a year post-intervention and were able to print and keep the therapy modules they completed online.

iCT-PTSD

iCT-PTSD is the internet-delivered version of cognitive therapy for PTSD, one of the TF-CBT programmes recommended by NICE (2018) and international treatment guidelines (ISTSS. 2019). The treatment builds on Ehlers and Clark's model of PTSD (2000). This model suggests that people with PTSD experience a sense of current internal (i.e., threat to sense of self) or external threat factors even though the trauma is in the past. Two sources drive this sense of current threat: excessively negative appraisals (personal meanings) of the trauma and/or its aftermath and a disjointed memory for the trauma that leads to easy triggering of intrusive memories that appear to happen in the "here and now". The problem is maintained as individuals use understandable, but unhelpful, cognitive strategies and behaviours to control the

perceived threat, which maintain the appraisals, memory characteristics and PTSD symptoms. The model suggests three treatment goals:

- Modify threatening appraisals (i.e., personal meanings) of the trauma and its sequelae
- Reduce reexperiencing by elaboration of the trauma memories and discrimination of triggers.
- Reduce cognitive strategies and behaviours that maintain the sense of current threat.

Core treatment procedures include:

- Individually tailored case formulation with the Ehlers and Clark model
- Reclaiming your life assignments
- Guided discovery and behavioural experiments to change the unhelpful appraisals and maintaining behaviours/ cognitive strategies
- Updating trauma memories (a three-step procedure involving accessing the worst moments (hot spots) in the trauma memory and their meanings through imaginal reliving or narrative writing, identifying information that updates the meanings, and actively linking the new information with the relevant moment in memory)
- Identification and discrimination of triggers (then versus now)
- Site visit
- Blueprint to summarise what the patient has learned in therapy

All these procedures are realised in the iCT-PTSD treatment modules (see Table S1). There are special pages where patients write or record the story of their trauma and later update its worst moments, a page to work on individual hot spots, and a page to upload memory triggers and practice the Then versus Now discrimination. Updating memories was mainly done by writing narratives of their traumatic experiences within the programme and written updates of meanings that were read out with emotional engagement, although participants also had the option to audio-record their trauma story.

Table S1 iCT-PTSD modules

The following modules are released to all participants in iCT-PTSD, as they represent core procedures of CT-PTSD:

1. Introducing the Treatment
2. Reclaiming your Life (with my Weekly Plan)
3. It's All Understandable
4. Updating your Memories – Part 1 – Telling the Story of your Trauma
5. Updating Your Memories - Part 2 - Finding your Hot Spots
6. Updating Your Memories - Part 3 - How to Update your Hot Spots
7. Updating Your Memories - Part 4 - Updating your Hot Spots
8. Spotting Memory Triggers
9. Beating Memory Triggers - Part 1 - THEN versus NOW
10. Beating Memory Triggers - Part 2 - THEN versus NOW practise using the My Triggers page
11. Beating Memory Triggers - Part 3 - Tackling Triggers in Everyday Life
12. Understanding and Dealing with Risk - Part 1
13. Understanding and Dealing with Risk - Part 2
14. My Site Visit
15. My Blueprint
16. Preparing for Your First Follow-up
17. Preparing for Your Second Follow-up
18. Preparing for Your Final Follow-up

In addition, the therapist can release the following optional modules, depending on the individual case formulation:

19. Rumination
20. Overcoming Shame and Humiliation
21. Dealing With Anger
22. Dealing With Guilt
23. Sleep
24. Dissociation
25. I am Physically Different Now
26. Earlier Memories
27. Childhood Trauma
28. Self-Esteem
29. Chronic Pain and PTSD
30. Death of a Loved One
31. Panic Attacks
32. Overcoming Depression
33. Managing Your Inner Critic
34. Dealing with Drugs and Alcohol

---

iStress-PTSD

The iStress stress management therapy programme developed by Andersson and colleagues (Persson Asplund et al., 2018, 2023) focuses on achieving a balance between different aspects of life (e.g., work, relaxation, social life) and teaching and practising a wide range of coping strategies that help patients regulate their emotions and cope with stressors. Therapists guide patients in identifying stressors in their lives and in learning and practising the stress management skills. They support them in applying the skills to whatever stressor the patient chooses to work on. iStress was adapted for people with PTSD and includes psychoeducation about PTSD, training in problem solving and training in techniques for stress reduction and coping with PTSD symptoms such as applied relaxation training, challenging irrational thoughts, mindfulness and training to improve sleep efficiency, as well as exposure to avoided situations. Treatment modules are listed in Table 2. Patients also work on challenge areas of their choosing such as coping with memories or worry. In contrast to iCT-PTSD, therapists do not steer patients to focus on their trauma or trauma memories. A wide range of stressors in their current lives is considered. However, therapists also do not steer the patient away from applying the coping skills to their trauma(s). Should patients choose to do this, therapists support them with trauma-focused applications of the stress management techniques.

Table S2: iStress-PTSD modules

The following modules will be released to all participants assigned to iStress-PTSD:

1. Introduction
2. About Stress – Part 1
3. About Stress – Part 2
4. In Balance – Part 1
5. In Balance – Part 2
6. Challenge your Thoughts – Part 1
7. Challenge your Thoughts – Part 2
8. Sleep and Mindfulness – Part 1
9. Sleep and Mindfulness – Part 2
10. Overcoming Challenges
11. Overcoming Challenges Continued
12. Plan your Time
13. Be Kind to Your Brain
14. Maintenance and Closure
15. Preparing for Your First Follow-up
16. Preparing for Your Second Follow-up
17. Preparing for Final Follow-up
18. For participants who dissociate, therapists release an additional module: Understanding Dissociation.

In addition, the participant completes a range of diaries (e.g., stress diary, relaxation diary, exposure diary) and chooses which of the following challenge areas connected to their PTSD they will work on (each of these is addressed in 3 to 4 modules):

19. Anger (3 parts)
20. Coping with Memories (3 parts)
21. Drugs and Alcohol (4 parts)
22. Pain (4 parts)
23. Worry (4 parts)

References

Ehlers, A, Clark, DM. A cognitive model of posttraumatic stress disorder. Behav Res Ther 2000; 38: 319-45. doi: 10.1016/s0005-7967(99)00123-0

International Society of Traumatic Stress Studies. Posttraumatic stress disorder prevention and treatment guidelines. 2019. Available from: http://www.istss.org/getattachment/Treating-Trauma/New-ISTSS-Prevention-and-Treatment-Guidelines/ISTSS_PreventionTreatmentGuidelines_FNL.pdf.aspx

National Institute for Health and Care Excellence. Posttraumatic stress disorder (PTSD). London: NICE. 2018. (Clinical Guideline 116). https://www.nice.org.uk/guidance/ng116

Persson Asplund, R., Asplund, S., von Buxhoeveden, H., Delby, H., Eriksson, K., Gerhardsson, M., Johansson, T., Palm, J., Torstensson, J., Ljótsson, B., Carlbring, P., & Andersson, G. Work-focused versus generic internet-based interventions for employees with stress-related disorders: Randomized controlled trial. J Med Internet Res 2023;25:e34446. doi: 10.2196/34446

Persson Asplund R, Dagöö J, Fjellström I, Niemi L, Hansson K, Zeraati F et al. Internet-based stress management for distressed managers: results from a randomised controlled trial. Occupat Environmental Med 2018;75:105-13. doi:10.1136/oemed-2017-104458

Wild J, Warnock-Parkes E, Grey N, Stott R, Wiedemann M, Canvin L, et al. Internet-delivered cognitive therapy for PTSD: a development pilot series. Eur J Psychotraumatol 2016; 31019. doi:10.3402/ejpt.v7.31019

Supplement 4: Reasons given for dropping out of treatment or follow-up (all allocated)

Withdrawal from Treatment	iCT-PTSD (n = 10)	iStress-PTSD (n =10)
Physical illness	1	0
Illness in family	2	0
Too busy/ unable to continue	4	2
Felt better	1	1
Felt treatment was too soon	0	2
Requested face-to-face/ immediate treatment/ started other treatment	0	3
Did not like some treatment components/preferred own strategies	0	2
No reason given	2	0
**Withdrawal from Follow-up**	**iCT-PTSD (n = 3)**	**iStress-PTSD (n = 3)**
Illness	1	0
Too busy	1	1
Relapse in PTSD symptoms	1	0
Started other treatment	0	1
COVID-related stressors	0	1

Note: 1 participant dropped out of the waitlist condition because they sought immediate treatment

Supplement 5: Treatment compliance, acceptability, credibility and satisfaction, working alliance, and time commitment

In both treatment conditions, the majority of participants completed the core treatment modules, and completion of pages per module was 77.2% for iCT-PTSD and 71.3% for iStress-PTSD. iCT-PTSD participants attended a similar number of weekly calls with the therapist as iStress-PTSD participants (9.8 vs 9.2, n.s.), and somewhat more calls during the booster phase of treatment (2.6 vs 2.2, p = .045). Overall, the therapist time per patient for phone calls and messaging was 6.7 hours (SD 3.0) in iCT-PTSD and 5.7 (SD 2.1) hours for iStress (p = .004). Participants receiving iCT-PTSD were logged into the programme, including for questionnaire completion, for 24.1 (SD 23.1) hours and 16.2 (SD 14.3) hours for iStress-PTSD (*p* = .004). This includes time for breaks away from the computer, which could not be recorded separately. This difference was in part due to iStress-PTSD participants downloading the audiorecordings with applied relaxation and mindfulness instructions onto their smartphones so that time listening to the recordings could not be recorded and completing the diaries offline rather than within the programme, and iCT-PTSD participants working on trauma narratives, including breaks.

Participants receiving iCT-PTSD reported significantly greater satisfaction with treatment, both in terms of the clinical service and the online treatment, than those receiving iStress-PTSD. Credibility and working alliance ratings were similar in both groups.

Variable			Immediate Allocation			All Allocated to Treatment	
		n	iCT-PTSD (n=92)	iStress-PTSD (n = 93)	n	iCT-PTSD (n = 107)	iStress-PTSD (n = 105)
Completed treatment		185			212		
	No		9 (9.8%)	7 (7.5%)		10 (9.3%)	10 (9.5%)
	Yes		83 (90.2%)	86 (92.5%)		97 (90.7%)	95 (90.5%)

Received minimum treatment dose		185			212		
	No		9 (9.8%)	5 (5.4%)		9 (8.4%)	9 (8.6%)
	Yes		83 (90.2%)	88 (94.6%)		98 (91.6%)	98 (91.4%)
Treatment credibility rating (patients, 0-30) [1].		151	24.40 (4.66)	23.67 (4.49)	169	24.52 (4.68)	23.70 (4.39)
Working Alliance Scale (patients, 2w, 12-84) [2].		148	76.79 (8.20)	74.55 (9.09)	165	77.05 (7.88)	74.70 (8.93)
Working Alliance Scale (therapists, 2w, 12-84) [2].		184	75.31 (8.02)	73.63 (7.00)	210	75.26 (7.97)	73.43 (7.01)
**Patient Experience Questionnaire (13w)** [3].							
Satisfaction with clinical service (0-4)		159	3.84 (0.35)	3.67 (0.52)	182	3.86 (0.33)	3.62 (0.64)
Satisfaction with online treatment (0-4)		159	3.59 (0.52)	3.35 (0.67)	182	3.60 (0.51)	3.31 (0.75)
**Therapist and patient time**							
Weekly calls (to 13w)		185	9.66 (3.36)	9.44 (2.40)	212	9.78 (3.31)	9.20 (2.68)
Booster calls (13w to 26w)		185	2.65 (1.39)	2.25 (1.33)	212	2.64 (1.38)	2.20 (1.34)
Total number of calls		185	12.32 (4.48)	11.69 (3.36)	212	12.42 (4.40)	11.40 (3.67)
Therapist time weekly calls (to 13w)		185	216.24 (105.04)	188.01 (63.99)	212	219.78 (106.44)	181.73 (70.37)
Therapist time for weekly messages (to 13w)		185	102.13 (50.31)	97.71 (40.53)	212	103.47 (48.82)	94.29 (43.69)
Therapist time booster calls (13 to 26w)		185	58.73 (44.75)	45.25 (31.78)	212	58.15 (43.72)	44.50 (31.75)

Therapist time messaging booster phase (13 w to 26w)		185	21.66 (15.40)	20.59 (14.66)	212	21.24 (15.33)	20.52 (15.40)
Total therapist time		185	398.76 (178.10)	351.56 (112.89)	212	402.64 (178.08)	341.05 (126.87)
Time patients logged in weekly treatment phase (to 13w)		185	1,207.75 (1,085.26)	893.98 (571.45)	212	1,170.22 (1,029.03)	855.23 (568.90)
Time patient logged in booster phase (13w to 26w)		185	281.25 (538.26)	118.37 (129.72)	212	263.10 (503.73)	114.00 (127.45)
Total time patient logged in		185	1,489.00 (1,467.17)	1,012.34 (665.24)	212	1,433.32(1,388.42)	969.23662.36)

1. Borkovec TD, Nau SD. Credibility of analogue therapy rationales. J Behav Ther Exp Psychiatry. 1972;3: 257-260.
2. Horvath AO, Greenberg LS. Development and validation of the Working Alliance Inventory. J Couns Psychol, Vol 36(2). 1989. p.223-233. doi:10.1037/0022-0167.36.2.223
3. The Improving Access to Psychological Therapies Manual: Appendices and helpful resources. 2018. Available from: https://www.england.nhs.uk/wp-content/uploads/2018/06/iapt-manual-resources-v2.pdf

Supplement 6: Maintenance of Treatment Effects: Comparisons between iCT-PTSD and iStress-PTSD on the primary and secondary outcome measures (all allocated to treatment).

				Results of linear mixed models			
Measure	Time	Unadjusted Mean (SD) [n]		Adjusted difference [95%CI], *p* value	Standardised between-group effect size [95%CI]	Standardised within-group effect size [95%CI]	
		iCT-PTSD	iStress-PTSD			iCT-PTSD	iStress-PTSD
PTSD severity	26w*	12.54 (12.61) [103]	20.92 (18.30) [103]				
(PCL-5)	39w	13.08 (14.83) [92]	19.19 (18.57) [93]	-5.66 [-9.81, -1.50], .0079	0.43 [0.11, 0.74]	2.36 [2.15, 2.56]	2.03 [1.82, 2.23]
	65w	13.90 (14.02) [95]	19.47 (18.02) [97]	-5.01 [-9.14, -0.88], .018	0.38 [0.07, 0.69]	2.31 [2.10, 2.51]	2.03 [1.83, 2.24]
PTSD severity	26w*	13.74 (15.31) [102]	22.09 (19.39) [102]				
(IES-R)	39w	14.43 (17.45) [92]	20.73 (20.54) [93]	-6.53 [-11.39, -1.67], .0087	0.44 [0.11, 0.77]	2.58 [2.36, 2.79]	2.15 [1.94, 2.37]
	65w	15.35 (17.22) [95]	20.95 (20.21) [97]	-5.58 [-10.41, -0.75], .024	0.38 [0.05, 0.71]	2.51 [2.30, 2.73]	2.17 [1.95, 2.38]
PTSD severity, assessor (CAPS-5)	26w*	12.83 (12.27) [96]	18.85 (14.51) [97]				
	39w	13.07 (12.02) [85]	17.92 (15.08) [86]	-4.04 [-7.67, -0.41], .029	0.42 [0.04, 0.79]	2.71 [2.46, 2.96]	2.28 [2.03, 2.52]
	65w	12.71 (11.31) [83]	16.68 (15.05) [84]	-3.48 [-7.13, 0.16], .061	0.36 [-0.02, 0.73]	2.73 [2.48, 2.98]	2.37 [2.12, 2.62]
Depression (PHQ-9)	26w*	5.16 (5.57) [103]	7.32 (6.32) [103]				
	39w	5.17 (5.39) [92]	7.70 (6.86) [93]	-2.37 [-3.93, -0.81], .0031	0.39 [0.13, 0.65]	1.27 [1.08, 1.46]	0.96 [0.77, 1.14]
	65w	5.14 (5.72) [94]	7.07 (6.69) [97]	-1.61 [-3.15, -0.06], .042	0.27 [0.01, 0.52]	1.26 [1.07, 1.45]	1.08 [0.89, 1.26]
Anxiety (GAD-7)	26w*	4.66 (5.01) [103]	6.96 (6.45) [103]				
	39w	4.90 (5.76) [92]	6.60 (6.12) [93]	-1.55 [-3.06, -0.04], .044	0.29 [0.01, 0.58]	1.38 [1.18, 1.59]	1.20 [0.99, 1.40]
	65w	4.82 (5.25) [94]	6.35 (5.84) [97]	-1.30 [-2.80, 0.20], .089	0.24 [-0.04, 0.53]	1.38 [1.18, 1.59]	1.26 [1.05, 1.46]
Disability	26w*	8.13 (8.89) [102]	10.45 (9.79) [103]				

(WSAS)	39w	7.13 (8.34) [92]	10.16 (10.16) [93]	-3.16 [-5.45, -0.87], .0070	0.37 [0.10, 0.63]	1.36 [1.16, 1.55]	0.99 [0.80, 1.18]
	65w	6.59 (7.55) [94]	9.89 (10.40) [97]	-3.26 [-5.53, -0.99], .0051	0.38 [0.11, 0.64]	1.40 [1.21, 1.59]	1.04 [0.85, 1.23]
Well-being (WHO-5)	26w*	14.63 (5.64) [95]	11.87 (5.94) [95]				
	39w	14.58 (5.78) [85]	12.37 (6.36) [82]	2.16 [0.63, 3.69], .0059	0.49 [0.14, 0.84]	1.50 [1.26, 1.74]	1.10 [0.86, 1.35]
	65w	13.64 (5.42) [81]	11.22 (6.28) [77]	2.49 [0.94, 4.04], .0018	0.56 [0.21, 0.91]	1.30 [1.06, 1.55]	0.85 [0.61, 1.10]
Quality of Life (Q-LES-Q)	26w*	54.21 (11.34) [95]	49.75 (11.43) [95]				
	39w	54.11 (11.55) [85]	50.21 (12.30) [82]	3.85 [1.01, 6.69], .0081	0.40 [0.10, 0.69]	1.21 [1.01, 1.42]	0.86 [0.66, 1.07]
	65w	53.17 (10.18) [81]	48.71 (11.16) [77]	4.65 [1.77, 7.53], .0017	0.48 [0.18, 0.78]	1.14 [0.94, 1.35]	0.71 [0.50, 0.92]
Sleep Disturbance (ISI)	26w*	8.14 (6.65) [101]	9.03 (6.21) [102]				
	39w	8.38 (7.11) [92]	8.85 (7.02) [92]	-0.12 [-1.78, 1.54], .885	0.02 [-0.23, 0.26]	0.99 [0.81, 1.16]	1.05 [0.88, 1.22]
	65w	7.95 (6.81) [94]	8.43 (7.23) [97]	0.29 [-1.35, 1.94], .726	0.04 [-0.20, 0.29]	1.02 [0.85, 1.19]	1.15 [0.98, 1.32]

Notes. 26w* = score at 26 weeks; if data was missing at 26 weeks for a participant, the 13-week score was used as their end of treatment score on the respective measure. All statistics are based on intent-to-treat analyses using linear mixed-effect models for all participants assigned to treatment. Adjusted mean differences based on linear mixed-effects models adjusted for baseline scores and stratification variables. Standardised effect sizes calculated using the baseline standard deviation of whole sample. Within-group effect sizes represent change from baseline in each group.

Supplement 7

Comparisons between each of the treatments and waitlist with usual NHS care on the primary and secondary outcome measures at 13 weeks

Measure	Time	Unadjusted Mean (SD) [n]			Adjusted difference [95%CI], *p* value		Standardised Between-Group Effect size [95%CI]		Standardised Within-Group Effect size [95%CI]		
		iCT-PTSD	iStress-PTSD	Wait	iCT-PTSD vs Wait	iStress-PTSD vs Wait	iCT-PTSD vs Wait	iStress-PTSD vs Wait	iCT-PTSD	iStress-PTSD	Wait
PTSD severity (PCL-5) [1]	Pre	45.80 (13.32) [92]	47.66 (12.39) [93]	45.88 (11.13) [32]							
	6w	27.50 (16.41) [86]	26.36 (15.85) [88]	41.22 (13.45) [27]	12.08 [6.43, 17.74], <.0001	14.26 [8.63, 19.89], <.0001	0.96 [0.51, 1.41]	1.13 [0.69, 1.58]	1.44 [1.22, 1.66]	1.67 [1.45, 1.89]	0.44 [0.05, 0.84]
	13w	15.52 (14.29) [86]	21.73 (17.13) [91]	36.68 (16.81) [31]	20.98 [15.50, 26.45], <.0001	16.19 [10.74, 21.63], <.0001	1.67 [1.23, 2.10]	1.29 [0.85, 1.72]	2.39 [2.17, 2.62]	2.07 [1.86, 2.29]	0.74 [0.36, 1.11]
PTSD severity (IES-R) [2]	Pre	54.34 (13.69) [92]	54.76 (13.75) [93]	51.66 (13.66) [32]							
	6w	29.86 (19.16) [84]	30.75 (18.09) [88]	45.37 (17.10) [27]	13.84 [7.03, 20.65], <.0001	14.21 [7.46, 20.95], <.0001	1.01 [0.51, 1.51]	1.04 [0.54, 1.53]	1.74 [1.50, 1.99]	1.73 [1.49, 1.97]	0.54 [0.11, 0.97]
	13w	15.99 (15.82) [84]	24.83 (19.75) [90]	39.97 (20.11) [31]	24.25 [17.64, 30.86], <.0001	16.56 [10.03, 23.09], <.0001	1.77 [1.29, 2.25]	1.21 [0.73, 1.69]	2.76 [2.51, 3.00]	2.19 [1.95, 2.43]	0.86 [0.45, 1.27]

PTSD severity, assessor (CAPS-5) [3]	Pre	41.01 (9.54) [92]	41.24 (9.39) [93]	41.03 (10.20) [32]							
	13w	14.53 (12.27) [78]	18.04 (14.14) [82]	32.57 (12.34) [30]	17.91 [13.86, 21.96], <.0001	15.06 [11.03, 19.09], <.0001	1.88 [1.45, 2.30]	1.58 [1.16, 2.00]	2.76 [2.49, 3.04]	2.40 [2.13, 2.67]	0.89 [0.43, 1.34]
Depression (PHQ-9) [4]	Pre	12.93 (5.85) [92]	13.75 (6.07) [93]	13.81 (5.84) [32]							
	6w	9.59 (6.42) [86]	9.10 (5.74) [87]	12.44 (6.60) [27]	2.08 [-0.13, 4.30], .065	2.74 [0.53, 4.94], .015	0.35 [-0.02, 0.73]	0.46 [0.09, 0.83]	0.56 [0.37, 0.76]	0.74 [0.55, 0.93]	0.27 [-0.07,0.61]
	13w	6.41 (5.84) [86]	8.09 (6.36) [91]	11.84 (6.49) [31]	5.02 [2.88, 7.16], <.0001	3.72 [1.60, 5.85], .0007	0.85 [0.49, 1.21]	0.63 [0.27, 0.99]	1.10 [0.91, 1.30]	0.96 [0.77, 1.15]	0.34 [0.02, 0.67]
Anxiety (GAD-7) [5]	Pre	12.51 (5.24) [92]	13.33 (5.39) [93]	12.56 (4.94) [32]							
	6w	8.82 (5.66) [84]	8.56 (5.45) [87]	12.30 (4.77) [27]	2.69 [0.69, 4.69], .0086	3.30 [1.31, 5.29], .0013	0.51 [0.13, 0.89]	0.63 [0.25, 1.01]	0.68 [0.48, 0.88]	0.87 [0.67, 1.06]	0.13 [-0.22, 0.48]
	13w	5.53 (5.01) [85]	7.38 (6.10) [91]	11.19 (5.33) [31]	5.53 [3.60, 7.46], <.0001	4.17 [2.26, 6.09], <.0001	1.05 [0.68, 1.42]	0.79 [0.43, 1.16]	1.33 [1.13, 1.53]	1.15 [0.95, 1.34]	0.28 [-0.05, 0.62]
Disability	Pre	18.86 (8.73) [92]	18.93 (8.72) [93]	18.98 (8.35) [32]							

(WSAS) [6]	6w	13.64 (9.01) [84]	15.21 (9.50) [87]	18.58 (9.27) [27]	4.38 [1.24, 7.52], .0065	3.06 [-0.07, 6.19], .055	0.51 [0.14, 0.87]	0.35 [-0.01, 0.72]	0.57 [0.38, 0.76]	0.40 [0.21, 0.59]	0.07 [-0.27, 0.40]
	13w	9.53 (8.92) [85]	12.51 (9.86) [91]	18.15 (8.98) [31]	8.59 [5.57, 11.60], <.0001	5.84 [2.85, 8.84], .0002	1.00 [0.65,1.34]	0.68 [0.33, 1.02]	1.08 [0.89, 1.27]	0.75 [0.56, 0.94]	0.10 [-0.22, 0.41]
Well-being (WHO-5) [7]	Pre	8.04 (4.34) [92]	7.14 (4.47) [93]	7.84 (4.21) [32]							
	6w	11.61 (5.39) [64]	10.42 (4.63) [72]	7.89 (4.66) [27]	-3.11 [-5.16, -1.06], .0031	-2.27 [-4.30, -0.25], .028	0.71 [0.24, 1.18]	0.52 [0.06, 0.98]	0.81 [0.55, 1.07]	0.70 [0.46, 0.95]	0.08 [-0.32, 0.49]
	13w	12.89 (5.93) [79]	11.80 (5.73) [82]	8.13 (4.78) [31]	-4.53 [-6.47, -2.59], <.0001	-4.10 [-6.04, -2.16], <.0001	1.03 [0.59, 1.48]	0.94 [0.49, 1.38]	1.07 [0.83, 1.31]	1.05 [0.81, 1.28]	0.07 [-0.32, 0.46]
Quality of Life (QOL) [8]	Pre	41.84 (9.42) [92]	41.23 (9.26) [93]	43.41 (9.76) [32]							
	6w	48.25 (11.67) [64]	47.83 (9.73) [72]	43.07 (10.51) [27]	-5.76 [-9.65, -1.88], .0039	-5.84 [-9.67, -2.01], .0030	0.61 [0.20, 1.03]	0.62 [0.21, 1.03]	0.67 [0.45, 0.89]	0.68 [0.47, 0.89]	0.01 [-0.36, 0.33]
	13w	51.88 (12.21) [79]	50.65 (11.40) [82]	45.48 (11.51) [31]	-7.35 [-11.03, -3.68], .0001	-6.79 [-10.44, -3.13], .0003	0.78 [0.39,1.17]	0.72 [0.33, 1.11]	1.04 [0.84, 1.25]	0.99 [0.79, 1.19]	0.22 [-0.11, 0.54]

Sleep Disturbance (ISI) [9]	Pre	14.95 (7.21) [92]	16.66 (6.28) [93]	16.66 (4.53) [32]							
	6w	10.69 (7.05) [81]	12.06 (7.07) [86]	16.26 (5.69) [27]	3.75 [1.26, 6.24], .0034	3.88 [1.42, 6.34], .0022	0.58 [0.19, 0.96]	0.60 [0.22, 0.97]	0.58 [0.38, 0.78]	0.69 [0.49, 0.88]	0.10 [-0.24, 0.45]
	13w	9.02 (7.31) [83]	9.70 (7.29) [89]	14.81 (6.12) [31]	4.48 [2.08, 6.89], .0003	5.13 [2.76, 7.50], <.0001	0.69 [0.32,1.06]	0.79 [0.42, 1.15]	0.86 [0.66, 1.05]	1.05 [0.86, 1.25]	0.29 [-0.04, 0.62]

Notes. Within-group effect sizes represent change from baseline in each group. All statistics are based on intent-to-treat analyses using linear mixed-effect models for immediate allocations. Adjusted mean differences based on linear mixed-effects models adjusted for baseline scores and stratification variablesAdjusted mean differences based on linear mixed-effects models adjusted for baseline scores and stratification variables. Standardised effect sizes calculated using the baseline standard deviation of whole sample.

1. Weathers FW, Litz BT, Keane TM, Palmieri PA, Marx BP, Schnurr PP. The PTSD Checklist for DSM-5 (PCL-5). Available from: http://www.ptsd.va.gov.
2. Weiss DS, Marmar CR. The Impact of Event Scale - Revised. In: Wilson J, Keane TM, editors. Assessing psychological trauma and PTSD. New York: Guilford; 1996. p. 399-411.
3. Weathers FW, Blake DD, Schnurr PP, Kaloupek DG, Marx BP, Keane TM. The Clinician-Administered PTSD Scale for DSM-5 (CAPS-5). 2015. Available from: http://www.ptsd.va.gov.
4. Kroenke K, Spitzer RL, Williams JBW. The PHQ-9. J Gen Intern Med. 2001;16(9): 606-13.
5. Spitzer Rl, Kroenke K, Williams JW, Löwe B. A brief measure for assessing generalized anxiety disorder: The GAD-7. Arch Intern Med. 2006;166(10): 1092-97
6. Mundt JC, Marks IM, Shear MK, Greist JH. The Work and Social Adjustment Scale: a simple measure of impairment in functioning. Br J Psychiatry. 2002; 180:461-64.
7. Topp CW, Ostergaard SS, Sondergaard S, Bech P. The WHO-5 well-being index: A systematic review of the literature. Psychother Psychosom. 2015;84:167-76.

8. Rapaport MH, Clary C, Fayyad R, Endicott J. Quality-of-life impairment in depressive and anxiety disorders. Am J Psychiatry. 2005;162(6): 1171-78.
9. Morin CM, Belleville G, Bélanger L, Ivers H. The Insomnia Severity Index: psychometric indicators to detect insomnia cases and evaluate treatment response. Sleep. 2011;34(5): 601-8.

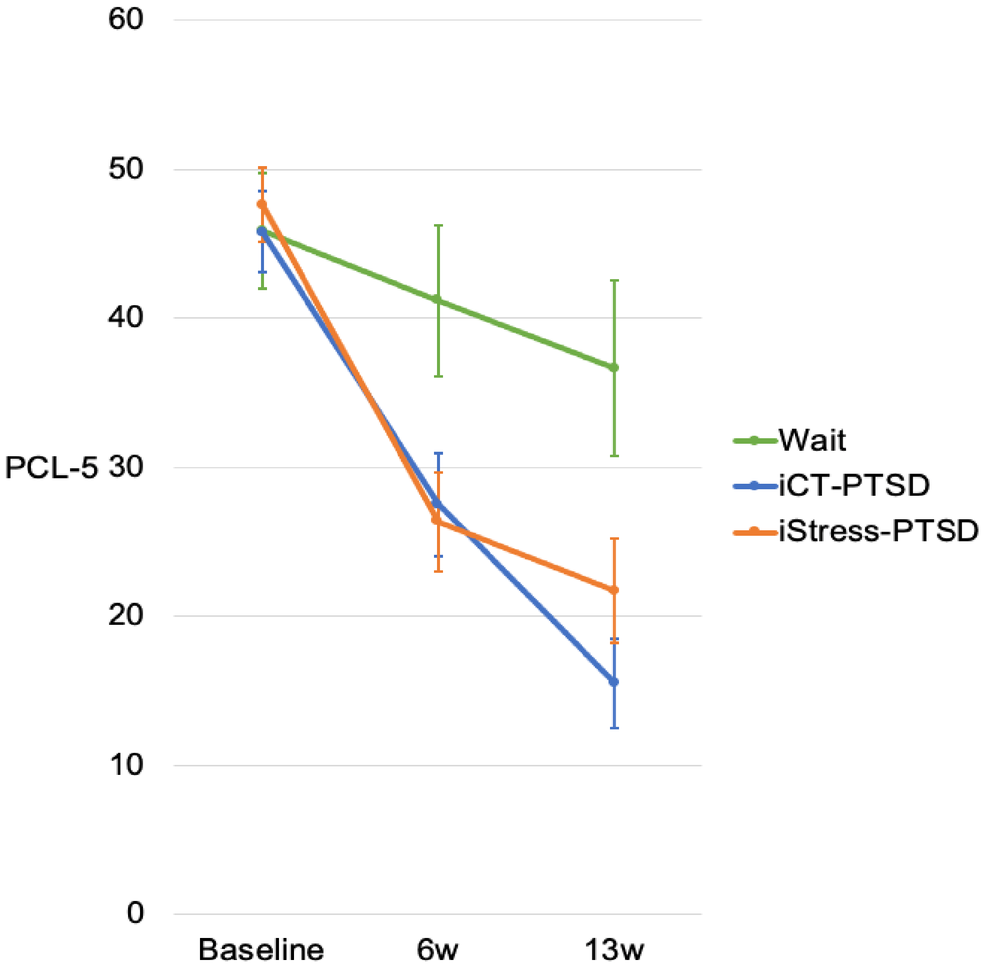

Supplement 7 Figure S7: Unadjusted means and 95% CIs for PTSD symptoms (PCL-5) for the comparisons of iCT-PTSD and iStress-PTSD with Waitlist with usual NHS care

Supplement 8: Dichotomous criteria for improvement and deterioration for comparisons of the treatments with wait with usual NHS care

		Comparisons of treatments with wait with usual NHS care (immediate allocation)		
**Criterion**	**Assessment**	**iCT-PTSD** [n]	**iStress** [n]	**WUC** [n]
**Self-reported PTSD symptoms**				
ITT clinically significant change on primary outcome (PCL-5) [1]	13w[10]	71% [65/92]	54% [50/93]	22 [7/32] [10]
	26w	76% [70/92]	58% [54/93]	
	39w	74% [68/92]	61% [57/93]	
	65w	71% [65/92]	58% [54/93]	
Asymptomatic on primary outcome (PCL-5) [2]	13w	45% [39/86]	31% [28/91]	7 [2/31]
	26w	58% [46/80]	43% [36/84]	
	39w	59% [46/78]	41% [34/83]	
	65w	58% [46/80]	43% [37/87]	
Worsening in PTSD symptoms (PCL-5) compared to baseline [3]	13w	0% [0/86]	1% [1/91]	10% [3/31]
	26w	0% [0/80]	4% [3/84]	
	39w	0% [0/78]	5% [4/83]	
	65w	1% [1/80]	3% [3/87]	

**Rater-assessed PTSD symptoms**				
No longer PTSD diagnosis (CAPS-5) [4]	13w[11]	85% [66/78]	71% [58/82]	13% [4/30] [11]
	26w	82% [63/77]	74% [58/78]	
	39w	88% [63/72]	70% [53/76]	
	65w	84% [58/69]	74% [55/74]	
Asymptomatic on CAPS-5 [5]	13w	46% [36/78]	35% [29/82]	3% [1/30]
	26w	58% [45/77]	41% [32/78]	
	39w	53% [38/72]	41% [31/76]	
	65w	54% [37/69]	47% [35/74]	
Worsening in CAPS-5 scores compared to baseline [6]	13w	0% [0/78]	5% [4/82]	0% [0/30]
	26w	1% [1/77]	3% [2/78]	
	39w	1% [1/74]	5% [4/76]	
	65w	3% [2/69]	4% [3/74]	
**Improving Access to Psychological Therapies (IAPT) criteria (PTSD and depression symptoms)**				
**IAPT recovery** [7]	Last assessment therapy/WUC[12]	79% [92]	66% [91]	19% [32] [12]
**IAPT reliable improvement** [8]	Last assessment therapy/WUC	93% [90]	87% [93]	39% [31]
**IAPT reliable deterioration** [9]	Last assessment therapy/WUC	0% [90]	3% [93]	7% [31]

[1] Defined as in Jacobson & Truax (1971] as (1) a reliable change on the main outcome measure (PCL-5 change ≥ 10 **and** (2) end of treatment PCL-5 score lower than 2 standard deviations below mean of treatment sample at baseline (i.e., PCL-5 < 21). ITT analysis; missing data scored as no clinically significant change, unless there was clear evidence from CAPS/ scores in online programme.

[2] Defined as PCL-5 ≤ 10. Calculated for participants with available data.

[3] Defined as worsening by 5 or more points on the PCL-5. Calculated for participants with available data.

[4] Defined as all DSM-5 PTSD criteria met with threshold of 2 for symptoms and interference ratings on CAPS-5 items. Calculated for participants with available data.

[5] Defined as CAPS-5 total ≤ 10. Calculated for participants with available data.

[6] Defined as worsening by 5 or more points on the CAPS-5. Calculated for participants with available data.

[7] Scoring according to the IAPT Manual v5 (https://www.england.nhs.uk/wp-content/uploads/2018/06/the-iapt-manual-v5.pdf): PCL-5 < 32 **and** PHQ-9 < 10; missing data scored as no recovery; denominator excludes patients with subthreshold PCL/PHQ scores at baseline

[8] Scoring according to the IAPT Manual v5 PCL change ≥ 10 **or** PHQ change ≥ 6, and no reliable deterioration in either measure; for cases with at least two data points; 4 cases had missing data (e.g., did not start treatment, missing questionnaire)

[9] Scoring according to the IAPT Manual v5: PCL change ≤ -10 **or** PHQ change ≤ - 6, and no reliable improvement in either measure; for cases with at least two data points; 4 cases had missing data (e.g., did not start treatment, missing questionnaire)

[10] Numbers needed to treat: iCT = 2.0; iStress = 3.1

[11] Numbers needed to treat: iCT = 1.4; iStress = 1.7

[12] Numbers needed to treat: iCT = 1.7; iStress = 2.1

References:

PCL-5: Weathers FW, Litz BT, Keane TM, Palmieri PA, Marx BP, Schnurr PP. The PTSD Checklist for DSM-5 (PCL-5). Available from: http://www.ptsd.va.gov.

CAPS-5: Weathers FW, Blake DD, Schnurr PP, Kaloupek DG, Marx BP, Keane TM. The Clinician-Administered PTSD Scale for DSM-5 (CAPS-5). 2013. Available from: http://www.ptsd.va.gov.

IAPT Manual: The Improving Access to Psychological Therapies Manual: Appendices and helpful resources. 2018. Available from: https://www.england.nhs.uk/wp-content/uploads/2018/06/iapt-manual-resources-v2.pdf

Jacobson NS, Truax P. Clinical significance: A statistical approach to defining meaningful change in psychotherapy research. J Consult Clin Psychol 1971;59(1):12–9.

Supplement 9: CACE analysis (complier-average casual-effect analysis)

A complier-average causal effect analysis (CACE) was used to test the influence of treatment compliance on the treatment effect. The R package AER (Kleiber & Zeileis, 2008) was used to compare each treatment against waitlist with usual NHS care, and the two treatments against each other. Outcome was the PCL-5 at 13 weeks.

Compliance with treatment was defined as per-protocol based on the minimum therapy to achieve benefits. This was operationalised as follows:

Patients in iCT-PTSD completed the following core procedures in the modules and/or phone calls:

- Treatment rationale and individual case formulation
- Reclaiming/rebuilding your life
- At least one of the core memory techniques: Updating memories and/or trigger discrimination

Patients in iStress-PTSD completed the following core procedures through the modules and/or phone calls:

- Treatment rationale and psychoeducation about stress and PTSD
- In balance and activity planning
- Learned least one of the core stress management techniques (applied relaxation, thought challenging, mindfulness, sleep efficiency)

Results

Comparison of treatments against WUC

The CACE result for the group difference between iCT and WUC was 22.30 [95% CI: 16.67, 27.92], which is similar to the group difference reported in Supplement 6 of 20.98 [95% CI: 15.50, 26.45].

The CACE result for the group difference between iStress and WUC was 17.74 [12.19, 23.28], which is similar to the group difference reported in Supplement 6 of 16.19 [95% CI: 10.74, 21.63].

Comparison between the treatment arms

Both analyses showed a group difference of around 5 points on the PCL-5.
iCT vs iStress = -5.08 [-9.26, -0.91] for the analysis of compliance with
(receiving the minimum dose of iCT); iStress vs iCT = 5.03 [0.79, 9.27] for the analysis of compliance with (receiving the minimum dose of) iStress. This is very similar to the difference of 5.15 [-9.21, -1.09] shown in Figure 3 for the linear mixed effects regression analysis.

Reference:

Kleiber C, Zeileis A. *Applied Econometrics with R*. Springer Verlag. (2008). https://CRAN.R-project.org/package=AER

Supplement 10: Adverse events and adversity during treatment

There were no serious treatment-related adverse events. Two iStress-PTSD participants (2%) reported a marked temporary increase in PTSD symptoms or dissociation that was judged treatment-related. Three further iStress-PTSD (3%), 2 iCT-PTSD (2%), and 1 WUC participant (3%) reported transitory increases in suicidal ideation, depression or self-harm that were judged as partially related to treatment/WUC.

Participants in both treatment arms also reported a range of other adversity during treatment (e.g., bereavement, serious medical illness (self or significant other), divorce, ongoing threats by perpetrators or new traumas) (iCT-PTSD: 109 events; iStress: 73 events). Some participants in both treatment arms reported negative effects of the COVID pandemic affected treatment (e.g., no privacy during calls, some treatment procedures not possible) or symptoms at the CAPS assessments (e.g., COVID self or significant other, masks reminder, loss of income), especially at the 1-year follow-up (3%, 9%, 10% and 20% at 13, 26, 39 and 65 weeks respectively).

Supplement 11: Moderation analyses

As part of the process evaluation, potential *moderators* were added to linear mixed effects regression models to test whether they interacted with treatment effects. A wide range of demographic (e.g., age, gender, ethnicity), clinical (e.g., comorbidity with major depression, anxiety disorders, dissociation, complex PTSD symptoms, substance use, self-referrals), and trauma-related characteristics (e.g., childhood trauma, number of traumas treated and number of trauma types experienced, permanent physical injury) were considered.

Moderators of treatment outcome

We examined a range of demographic (e.g., age, gender, ethnicity), demographic (e.g., age, gender, ethnicity), clinical (e.g., comorbidity with major depression, anxiety disorders, dissociation, complex PTSD symptoms, substance use, self-referrals), and trauma-related characteristics (e.g., childhood trauma, number of traumas, permanent physical injury) as possible moderators of treatment outcome. Severity of complex PTSD symptoms (ITQ]) and the severity of dissociation at baseline (TSDQ) showed significant moderation relationships in linear mixed effects models, but none of the other variables. The advantage of iCT-PTSD over iStress-PTSD was greater for participants scoring high on these scales compared to those with lower scores. The results contradict Gerger et al.'s (2014) suggestions to use nonspecific interventions for more complex presentations of PTSD.

References

ITQ Hyland P, Shevlin M, Brewin CR, Cloitre M. Downes AJ, Jumbe S, et al. Validation of posttraumatic stress disorder (PTSD) and complex PTSD using the International Trauma Questionnaire. Acta Psychiatr Scand 2017;136: 313-22.

TDSQ Murray J, Ehlers A, Mayou RA. Dissociation and posttraumatic stress disorder: Two prospective studies of road traffic accident victims. Br J Psychiatry 2002;180: 363-68.

Gerger H, Munder T, Barth J. Specific and nonspecific psychological interventions for PTSD symptoms: A meta-analysis with problem complexity as a moderator. J Clin Psychology 2014; 70(7): 601-15.

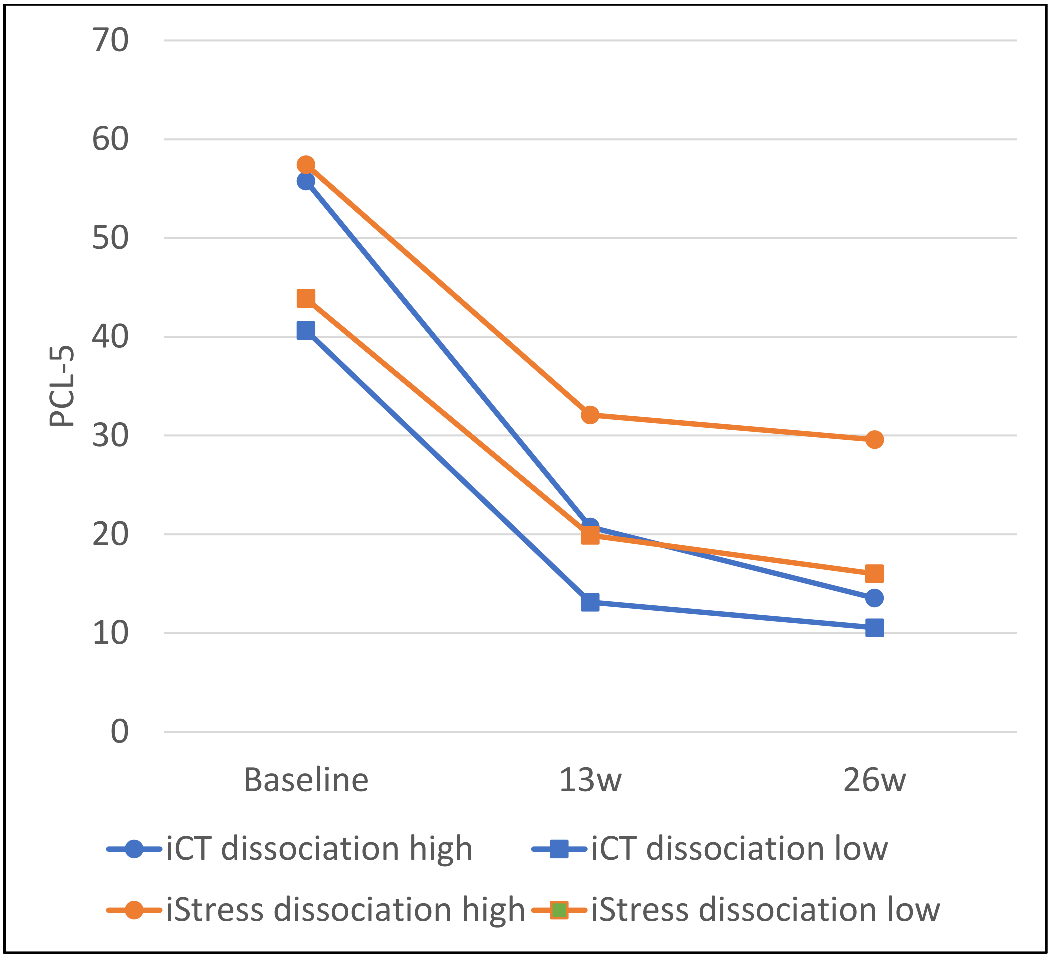

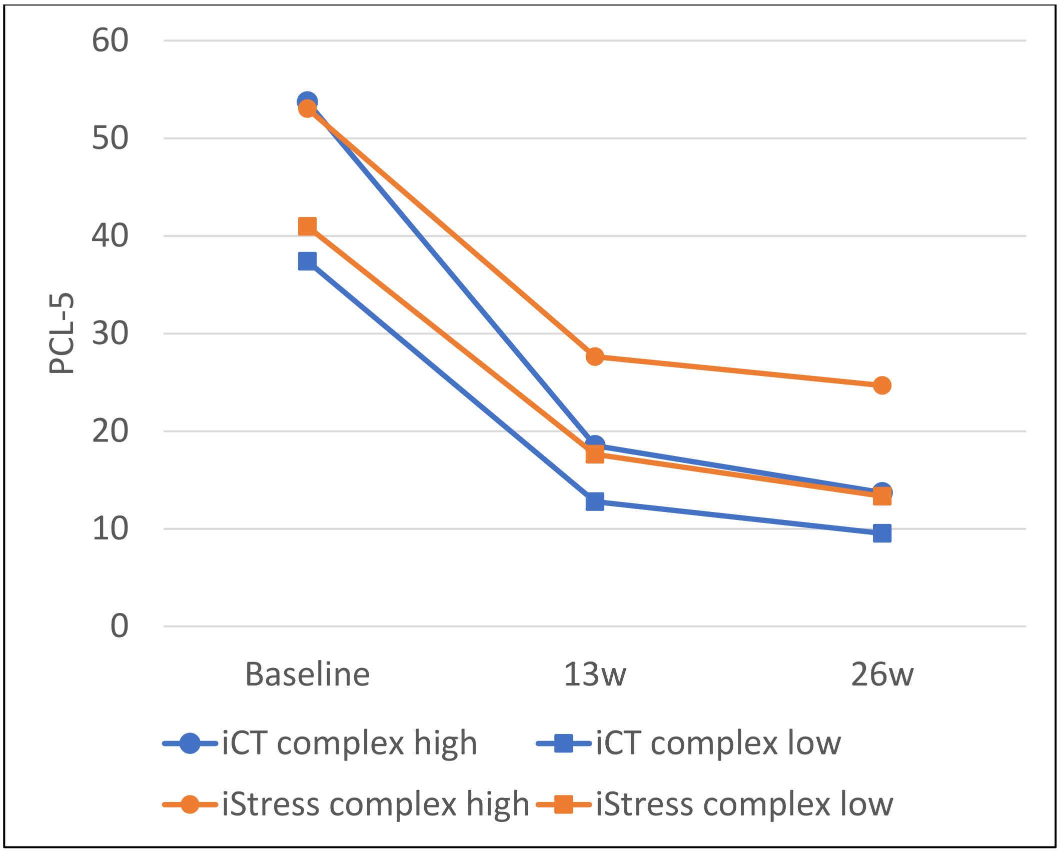

Moderation of differential treatment effects on PTSD symptoms (PCL-5). For participants high in dissociation or who met self-reported criteria for complex PTSD criteria differences between iCT-PTSD (blue) and iStress-PTSD (orange) were greater than for those with lower dissociation scores or complex PTSD symptoms, respectively

Supplement 12: Mediation Analyses

Method: Process measures for mediation analysis included measures of the cognitive factors hypothesized to maintain PTSD and targeted in CT-PTSD according to Ehlers and Clark's (2000) model of PTSD:

- Unhelpful appraisals of the trauma and its aftermath (short version of the Posttraumatic Cognitions Inventory (PTCI) (Foa et al. 1999)
- characteristics of trauma memories (Trauma Memory Questionnaire (TMQ) (Halligan et al. (2003), subscales Poor recall, Disjointedness and Flashback Quality),
- maintaining cognitive and behavioural coping responses (Response to Intrusion Questionnaire (RIQ) (Clohessy & Ehlers, 1999), Safety Behaviours Questionnaire (SBQ) (Dunmore et al. 2001), Trait-State Dissociation Questionnaire (TSDQ) (Murray et al. 2002).

In addition, a general measure of perceived coping ability was also given as a candidate mediator especially relevant for a coping-focused treatment such as iStress-PTSD (Generalized Self Efficacy Scale (GSES) (Schwarzer et al. 1995).

The role of process variables that may *mediate* differential clinical outcomes between each treatment and WUC were examined using linear mixed-effects models. The approach followed Freeman et al. (2022) using the principles of Baron and Kenny (1986) but using linear mixed effects models at each step. The approach involved four steps and three separate model fits. In two separate linear mixed effects models, the intervention was shown to be correlated with the outcome and then with the mediator. We then fitted the data to a third model with the outcome as the response and both the intervention and mediator as covariates. The parameters were extracted as per Baron and Kenny to obtain the total, direct, and indirect effects, and finally the percentage mediation was determined. The 6-week scores on each process variable were examined as a candidate mediator of the relationship between randomisation (each treatment vs. wait) and 13-week scores on the PCL-5. All models included baseline scores on the PCL-5 and mediator as covariates, plus the stratification variables of site and time since trauma. Separate models were used to compare the two active treatments directly, using the larger final allocation sample. These examined the 13-week process variable scores for the mediator, and 26-week PCL-5 scores as the outcome variable.

Table S12a shows the results of the mediation analyses of differences between the treatment conditions after the end of treatment (26 weeks). Significant indirect effects were found for the key processes specified in Ehlers and Clark's model of PTSD (2000): negative appraisals of the trauma and its aftermath (PTCI), unhelpful strategies to deal with intrusions (RIQ, rumination, thought suppression and numbing), safety behaviours (SBQ), disjointed trauma memories and flashback quality of intrusions (TMQ). Table S12b shows the mediation analyses of the differences between each of the treatments and WUC at 13 weeks, respectively. For both treatment conditions, the superiority compared to WUC was mediated by the similar cognitive mechanisms (negative appraisals, unhelpful coping strategies, disjointed memories) and dissociation. In contrast, general self-efficacy was not a mediator of treatment effects.

References:

Baron R, Kenny DA. The moderator-mediator variable distinction in social psychological research. J Pers Soc Psychol. 1986; 5: 1173–82.

Clohessy S, Ehlers A. PTSD symptoms, response to intrusive memories, and coping in ambulance service workers. Br J Clin Psychol. 1999;38: 251-65.

Dunmore E, Clark DM, Ehlers A. Cognitive factors involved in the onset and maintenance of PTSD. Behav Research and Ther. 1999;37: 809-29.

Ehlers, A, Clark, DM. A cognitive model of posttraumatic stress disorder. Behav Res Ther 2000; 38: 319-45. doi: 10.1016/s0005-7967(99)00123-0

Foa EB, Ehlers A, Clark DM, Tolin D, Orsillo S. The Post-traumatic Cognitions Inventory (PTCI). Development and validation. Psychol Assess. 1999;11: 303-14.

Freeman D, Lambe S, Kabir T, Petit A, Rosebrock L, Yu LM, et al.; gameChange Trial Group. Automated virtual reality therapy to treat agoraphobic avoidance and distress in patients with psychosis (gameChange): a multicentre, parallel-group, single-blind, randomised, controlled trial in England with mediation and moderation analyses. Lancet Psychiatry 2022; 9(5):375-88.

Halligan SL, Michael T, Clark DM, Ehlers A. Posttraumatic stress disorder following assault: the role of cognitive processing, trauma memory, and appraisals. J Consult Clin Psy 2003;71: 419-31.

Murray J, Ehlers A, Mayou RA. Dissociation and posttraumatic stress disorder: Two prospective studies of road traffic accident victims. Br J Psychiatry. 2002;180: 363-68.

Schwarzer R, Jerusalem M. Generalized Self-Efficacy Scale. In: Weinman J, Wright S, Johnston M. Measures in health psychology: A user's portfolio. Causal and control beliefs. Windsor, UK: NFER-NELSON; 1995. p. 35-7.

Table S12a. Mediation analyses of differences between iCT-PTSD and iStress-PTSD in PTSD symptoms (PCL-5 scores) at end of all treatment (26 weeks)

	Total Effect		Direct Effect		Indirect Effect		% Mediated
**Process Variable**	Adjusted Difference (SE) [95%CI]	*P*	Adjusted Difference (SE) [95%CI]	*p*	Adjusted Difference (SE) [95%CI]	*P*	
Trauma Appraisals (PTCI)	-7.37 (1.95) [-11.23, -3.52]	<.001	-4.48 (1.43) [-7.31, -1.65]	.002	2.70 (1.32) [0.11, 5.30]	.041	37
Responses to Intrusions (RIQ)	-7.51 (1.97) [-11.39, -3.63]	<.001	-2.10 (1.39) [-4.85, 0.65]	.134	5.05 (1.45) [2.22, 7.88]	<.001	67
Dissociation (TSDQ)	-7.47 (1.96) [-11.35, -3.60]	<.001	-4.55 (1.51) [-7.54, -1.56]	.003	2.04 (1.29) [-0.49, 4.57]	.113	
Safety Behaviours (SBQ)	-7.41 (1.94) [-11.24, -3.58]	<.001	-3.54 (1.57) [-6.64, -0.44]	.025	3.93 (1.18) [1.62, 6.23]	.001	53
Trauma Memory Qualities (TMQ)							
Poor Recall	-7.30 (1.96) [-11.17, -3.43]	<.001	-6.97 (1.87) [-10.65, -3.29]	<.001	0.05 (0.58) [-1.10, 1.19]	.937	
Disjointed	-7.39 (1.95) [-11.24, -3.53]	<.001	-4.90 (1.66) [-8.18, -1.63]	<.004	2.35 (1.08) [0.24, 4.47]	.029	32
Flashback	-7.39 (1.96) [-11.26, -3.52]	<.001	-2.45 (1.37) [-5.16, 0.25]	.076	5.05 (1.43) [2.26, 7.85]	<.001	68
Self-Efficacy (GSES)	-7.35 (1.97) [-11.22, -3.47]	<.001	-6.49 (1.78) [-10.00, -2.98]	<.001	0.63 (1.05) [-1.42, 2.68]	.547	

Notes. All models represent the iCT-PTSD vs iStress-PTSD comparison for all allocated participants. Mediator = Process variable at 13 weeks, Outcome = PCL-5 score at 26 weeks. All coefficients estimated using linear mixed effects models, with total, direct, and indirect effects calculated following Baron and Kenny [42]. Models included baseline scores on the mediator and outcome variables, site, and time since trauma as fixed covariates, and a random effect of participant. PTCI = Posttraumatic Cognitions Inventory; RIQ = Response to Intrusions Questionnaire; TSDQ = Trait State Dissociation Questionnaire; SBQ = Safety Behaviours Questionnaire; TMQ = Trauma Memory Questionnaire; GSES = General Self Efficacy Scale.

Table S12b: Mediation analyses of differences in PCL-5 scores at 13 weeks between each treatment and WUC (wait with usual NHS care)

Process Variable	Total Effect Adjusted Difference (SE) [95%CI]	*p*	Direct Effect Adjusted Difference (SE) [95%CI]	*p*	Indirect Effect Adjusted Difference (SE) [95%CI]	*P*	% Mediated
**PTCI**							
iCT-PTSD vs Wait	21.35 (2.79) [15.85, 26.86]	<.001	18.97 (2.43) [14.16, 23.77]	<.001	-4.38 (1.73) [-7.77, -0.98]	.011	20
iStress vs Wait	16.55 (2.77) [11.08, 22.02]	<.001	13.30 (2.42) [8.53, 18.08]	<.001	-4.49 (1.72) [-7.86, -1.11]	.009	27
**RIQ**							
iCT-PTSD vs Wait	21.18 (2.80) [15.65, 26.71]	<.001	17.63 (2.63) [12.45, 22.82]	<.001	-5.40 (1.53) [-8.40, -2.39]	<.001	25
iStress vs Wait	16.34 (2.78) [10.86, 21.81]	<.001	13.86 (2.57) [8.79, 18.93]	<.001	-3.98 (1.47) [-6.85, -1.10]	.007	24
**TSDQ**							
iCT-PTSD vs Wait	20.90 (2.78) [15.43, 26.38]	<.001	18.18 (2.50) [13.25, 23.11]	<.001	-5.23 (1.69) [-8.54, -1.92]	.002	25
iStress vs Wait	16.06 (2.76) [10.61, 21.50]	<.001	11.35 (2.51) [6.40, 16.30]	<.001	-6.32 (1.71) [-9.67, -2.96]	<.001	39
**SBQ**							
iCT-PTSD vs Wait	20.97 (2.76) [15.53, 26.41]	<.001	20.39 (2.56) [15.34, 25.43]	<.001	-2.29 (1.44) [-5.12, 0.54]	.113	
iStress vs Wait	16.30 (2.74) [10.89, 21.71]	<.001	15.37 (2.55) [10.34, 20.40]	<.001	-2.50 (1.44) [-5.33, 0.32]	.082	
**MQ Poor Recall** iCT-PTSD vs Wait	21.01 (2.78) [15.53, 26.49]	<.001	22.47 (2.81) [16.92, 28.01]	<.001	-0.93 (0.70) [-2.29, 0.44]	.183	
iStress vs Wait	16.35 (2.77) [10.90, 21.80]	<.001	16.89 (2.82) [11.32, 22.46]	<.001	-1.67 (0.78) [-3.19, -0.14]	.032	10
**MQ Disjointed** iCT-PTSD vs Wait	20.96 (2.78) [15.48, 26.45]	<.001	20.01 (2.67) [14.74, 25.28]	<.001	-3.12 (1.24) [-5.55, -0.68]	.012	15
iStress vs Wait	16.20 (2.76) [10.75, 21.65]	<.001	13.56 (2.70) [8.24, 18.88]	<.001	-4.48 (1.32) [-7.06, -1.91]	.001	28
**MQ Flashback** iCT-PTSD vs Wait	21.11 (2.79) [15.60, 26.62]	<.001	19.98 (2.38) [15.29, 24.68]	<.001	-2.68 (1.74) [-6.10, 0.74]	.124	
iStress vs Wait	16.30 (2.77) [10.83, 21.77]	<.001	14.09 (2.38) [9.40, 18.78]	<.001	-3.28 (1.74) [-6.69, 0.13]	.060	
**GSES**							
iCT-PTSD vs Wait	20.92 (2.78) [15.44, 26.40]	<.001	22.06 (2.69) [16.75, 27.37]	<.001	-2.23 (1.33) [-4.84, 0.38]	.094	
iStress vs Wait	16.27 (2.77) [10.82, 21.72]	<.001	17.45 (2.64) [12.23, 22.66]	<.001	-1.91 (1.31) [-4.46, 0.65]	.144	

Notes. All models represent the iCT-PTSD vs Wait or iStress-PTSD vs Wait comparsions comparison for immediate allocations. Mediator = Process variable at 6 weeks, Outcome = PCL-5 score at 13 weeks. All coefficients estimated using linear mixed effects models, with total, direct, and indirect effects calculated following Baron and Kenny's principles [42], adapted for longitudinal analyses as in [43]. Models included baseline scores on the mediator and outcome variables, site, and time since trauma as fixed covariates, and a random effect of participant. PTCI = Posttraumatic Cognitions Inventory; RIQ = Response to Intrusions Questionnaire; TSDQ = Trait State Dissociation Questionnaire; SBQ = Safety Behaviours Questionnaire; TMQ = Trauma Memory Questionnaire; GSES = General Self Efficacy Scale

Supplement 13:
Exploratory analysis: Trauma-focused use of exposure in iStress-PTSD

An exploratory analysis examined whether the degree of trauma-focused exposure undertaken within iStress-PTSD was associated with clinical outcomes on the primary outcome measure (PCL-5) at 13 and 26 weeks. The degree of exposure was rated by therapists at the end of treatment on the basis of exposure hierarchies the patient had worked on from no trauma-related exposure (0), some part (1), a significant part (2) or nearly all (3) of trauma-related hierarchy completed. A linear mixed effects model for the iStress-PTSD condition showed that the degree of trauma-focused exposure showed a significant linear relationship with decrease in PCL-5 scores across treatment (estimate = -8.58, SE = 2.69, p = .002). A greater degree of trauma-focused exposure was associated with lower PCL-5 scores at 13 and 26 weeks.

This exploratory analysis further supports the conclusion that trauma-focus enhances outcomes: participants who exposed themselves to reminders of the trauma did better than those who worked on other hierarchies of avoided situations.

Supplement 14: Themes in open responses, *Patient Experience Questionnaire*

**iCT-PTSD**	**iStress-PTSD**
**Effect on Life**	**Effect on Life**
'It has completely changed my life for the better'	*'I really noticed an improvement and am more able to cope with life'*
'Absolutely fantastic – this is life saving and relationship saving stuff'	*'..given me my life quality back'*
'It has changed the way I deal with my trauma and has given me my life back'	*'Helped me to come to terms with my trauma'*
'It has helped me turn my life around in a matter of months and have hope again when I thought I wouldn't have any. It has gotten me to a good place in my life again.'	*'It helps manage day-to-day life'*
'This really helped me put my trauma behind me and I'm finally moving on with my life and so happy'	*'Has changed my life generally'*
**Therapist support**	**Therapist support**
'with help and communication with the amazing therapist I managed to complete all the modules which has completely changed me as a person'	
'Speaking to the therapist was really helpful.'	*'loved the commitment, help and understanding'*
'My therapist has been incredibly helpful in helping me overcome my problems'	*'speaking with my therapist was helpful'*
'It has been excellent, I really feel I have been looked after'	*'I really appreciate the phone conversations'*
'wonderful therapist, I could not have done it without her'	*'I found ... having my therapist on hand to talk to in any areas of difficulty a real help.*
**Content of programme**	**Content of programme**
'I found different ways of seeing and reacting to things'	*'Good programme that covers a very wide variety of issues and strategies, very relevant'*
'I have felt that my mind has been systematically ordered. This has helped'	*'Feeling so much better.. down to me adopting relaxation'*
'I have learnt techniques that I will continue to use'	*'Brilliant in helping me with my sleep and managing my anxiety'*
'It has been great to learn coping strategies and acceptance of the trauma'	*'It has given me tools to manage my anxiety, fear and worry'*

'It has been helpful and very very informative and shed a lot of light on what I have been going through.'	*'Kind caring and effective'*
'It has been hard work and stressful but it is giving me the tools to put myself back together.'	*'did not think this would work but gave it a go and it was amazing how its allowed me to understand myself and educate me on stressors not just regarding ptsd but also challenges life will throw at you'*
**Online treatment**	**Online treatment**
'I have enjoyed the feeling of personal progress whilst using the online treatment. I feel more in control'	*'It was easy to log in wherever and whenever I wanted'*
'It has been really helpful having the flexibility of an online programme I can log into when I feel like it'	*'It was helpful that I could sit and think about the answers'*
'Accessible and rewarding'	*'working ...online was the best way to fit around my current lifestyle'*
'It was very good. Did underestimate online therapy, didn't think it was going to work at all but it was probably the best thing I did'	*'The online portion definitely meant I was able to engage with it much more effectively. I don't know how useful the service was overall to me, but the method of delivery really helped'.*
**Found less helpful**	**Found less helpful**
'two-factor authentification'	*'diaries very time-consuming'*
'amount of questions in the assessments'	*'too much emphasis on questionnaires'*
'Colour scheme has been a hinderance'	*'a bit too general'*
'modules helpful, but sometimes difficult to use'	*'I have learned some good coping skills for panic but I still have nightmares'*
'can be time consuming'	*'struggled with the daily amount of time needed'*

Reference:

The Improving Access to Psychological Therapies Manual. Appendices and helpful resources. 2018. https://www.england.nhs. uk/wp-content/uploads/2018/06/iapt-manual-resources-v2.pdf

Supplement 15: Primary outcome (PCL-5) by gender*

(Unadjusted Mean (SD) [n], all allocations)

Time point	iCT-PTSD		iStress-PTSD	
	Female	Male	Female	Male
Pre	45.61 (13.98) [79]	43.39 (13.13) [28]	47.75 (12.54) [75]	44.29 (13.34) [28]
6w	26.97 (16.68) [72]	26.19 (16.34) [26]	26.22 (15.49) [70]	27.54 (17.84) [24]
13w	15.12 (12.88) [73]	16.19 (16.95) [27]	22.19 (17.37) [74]	23.12 (18.00) [25]
26w	10.96 (11.36) [67]	12.73 (12.91) [26]	17.78 (16.49) [67]	20.15 (17.99) [26]
39w	13.47 (15.41) [70]	11.82 (12.91) [22]	19.71 (19.04) [68]	16.04 (16.31) [23]
65w	13.80 (13.96) [72]	14.22 (14.53) [23]	19.21 (18.07) [71]	17.08 (15.09) [24]

Note: 2 participants reported identifying with other gender identities. Their scores were not included to protect anonymity.

Supplement 16: Changes in psychotropic medication during treatment/wait

	iCT-PTSD (n=92)	iStress (n=93)	Wait with usual NHS care (n=32)
**During weekly treatment**			
Started or restarted psychotropic medication	3	3	0
Increased dose	1	1	1
Decreased dose	0	0	0
Fluctuating doses	1	0	0
Stopped	0	0	0
Changed medication	0	0	0
**During booster period**			
Started new psychotropic medication	1	3	N/A
Increased dose	1	1	N/A
Decreased dose	1	0	N/A
Fluctuating doses	0	0	N/A
Stopped	2	0	N/A
Changed medication	1	0	N/A

Accessed other forms of psychological interventions during treatment

	iCT-PTSD (n=92)	iStress (n=93)	Wait with usual NHS care (n=32)
**During weekly treatment**			
Started face-to-face psychological therapy for PTSD	0	1	1
Assessed by other service	1	0	0
Started therapy for other mental health issues	0	0	0
Counselling for other issues	0	1	0
Used mental health apps	0	2	0
**During booster period**			
Started face-to-face psychological therapy for PTSD	0	1	N/A
Assessed by other service	1	0	N/A
Counselling for other issues	2	0	N/A
Used mental health apps	0	0	N/A

Statistical Analysis Plan

Trial Full Title: A randomised controlled trial of therapist-assisted online psychological therapies for post-traumatic stress disorder

Internal Reference Number / Short title: STOP-PTSD

Ethics Ref: 17/WM/0441

IRAS Project ID: 224759

ISRCTN Number: 16806208

NIHR CRN Portfolio: 36855

Principal Investigator: Professor Anke Ehlers

Trial Statistician and SAP Author: Dr Esther Beierl

Senior Statistician: Professor Rafael Perera-Salazar

Protocol Version: 3 (26th September 2019)

SAP Version: 1 (11th November 2021

Statistical Analysis Plan

Version history

SAP Version 1, 11th November 2021, Protocol version 3 - 26th September 2019

Final version: …

Statistical Analysis Plan

Version 1.0

Chief Investigator: Professor Anke Ehlers

Date: 11th November 2021

Signature:

Trial Statistician and SAP Author: Dr. Esther Beierl

Date: 11th November 2021

Signature:

Senior Statistician: Professor Rafael Perera-Salazar

Date: 24 November 2021

Signature:

Statistical Expert, Trial Oversight Committee: Professor Kerry Hood

Date: 24th November 2021

Signature:

A randomised controlled trial of therapist-assisted online psychological therapies for post-traumatic stress disorder (STOP-PTSD) – Statistical Analysis Plan (SAP)

1. Introduction

1.1. Background and rationale of the trial

Posttraumatic stress disorder (PTSD) is a common disorder with an estimated 12- month prevalence of 1.3 to 3.6% (NICE, 2005).

Trauma-focused cognitive behavioural therapies are recommended as first-line treatments for PTSD by the NICE guidelines (NICE, 2005, 2018). A Cochrane review (Bisson, Roberts, Cooper, & Lewis, 2013) proposed that nontrauma-focused cognitive behavioural therapies are similarly effective (at least in the short-term) and are superior to other therapies and wait-list conditions. Direct comparisons of trauma-focused (such trauma-focused cognitive behavioural therapies for PTSD) and non-trauma focused therapies (such as stress-management) for the treatment of PTSD are needed.

Although treatment options for PTSD have improved over the last decades, many people suffering from PTSD are not able to receive effective psychotherapy due to a range of factors (shortage of psychotherapists, living far away from treatment centres, being unable to go to psychotherapy during working hours, etc.). Therefore, more efficient forms of treatment delivery, such as therapist-assisted online psychological treatments as an alternative to face-to-face therapy (therapist contact is remote and less time is needed to treat a patient), are under development and first studies have shown evidence for efficacy (Wild et al., 2016; Ivarsson et al., 2014; Knaevelsrud & Maercker, 2007; Spence et al., 2011).

The randomised controlled trial will compare a novel trauma-focused therapist-assisted online psychological therapy (internet-based cognitive therapy for PTSD, iCT-PTSD, Wild et al., 2016) and a comprehensive non-trauma-focused therapist-assisted online psychological therapy (internet-based stress management therapy, iStress-PTSD; Persson Asplund et al., 2017). Both treatments will be compared with a wait-list to control for the natural recovery that is sometimes seen in PTSD samples.

iCT-PTSD (Wild et al., 2016) implements the therapeutic procedures of cognitive therapy for PTSD (Ehlers et al., 2005), a trauma-focused cognitive-behavioural treatment that is based on Ehlers and Clark’s (2000) model of PTSD. It aims to change negative appraisals about the traumatic event and/or its consequences, updating the worst moments of traumatic memories with less threatening meanings, and reducing maladaptive behaviours and cognitive strategies that maintain symptoms.

Stress management is a nontrauma-focused cognitive behavioural treatment for PTSD that teaches strategies to cope with stressors in everyday life and the symptoms of PTSD. The trial uses the internet version of iStress, a very comprehensive modular stress management therapy programme (Zeterqvist et al., 2003; Persson Asplund et al., 2017), that was translated into English and adapted for PTSD.

1.2. Trial objectives

Primary research question

(1) Is iCT-PTSD more efficacious than iStress-PTSD, does it lead to greater PTSD symptom reduction compared to iStress-PTSD?

Other research questions

(2) Does iCT-PTSD lead to greater improvement in depression, anxiety, well-being, disability, quality of life, and sleep problems than iStress-PTSD?

(3) Are iCT-PTSD and iStress-PTSD efficacious, do they lead to greater symptom improvement in PTSD symptoms than a wait-list condition?

(4) Do iCT-PTSD and iStress-PTSD lead to greater depression, anxiety, well-being. disability, quality of life and sleep problems than a 13-week wait-list condition?

(5) Is iCT-PTSD more cost-effective than iStress-PTSD, is the cost per patient with a clinical improvement in PTSD symptoms and costs per QALY smaller? (analysis performed and reported by Dr. Tsiachristas, covered by separate health economics analysis plan)

(6) Do changes in PTSD-specific (appraisals, memory, maintaining strategies) and non-specific factors (alliance, self-efficacy) mediate improvement in PTSD symptoms with iCT-PTSD and iStress-PTSD?

(7) How do patients describe their experience with using online psychological treatment?

(8) Are the clinical effects of iCT-PTSD and iStress-PTSD maintained during long-term follow-up?

Exploratory research question

(9) Within the iStress-PTSD condition: Do patients have better outcomes if their exposure practice in therapy is trauma-related?

10) Do demographic or trauma characteristics or comorbidity affect treatment response?

2. Trial methods

2.1. Design

The design is a single blind randomised controlled trial comparing two therapist-assisted internet-based psychological treatments for post-traumatic stress disorder and a wait-list condition (superiority trial), with an embedded process study.

2.2. Randomisation

Eligible participants who consent to participate in the trial are randomised by an online programme designed by the Primary Care CTU, University of Oxford, to one of the three trial conditions (iCT, iStress, wait-list) at a 3:3:1 ratio using minimisation with randomisation (chance of selecting minimisation group 80%). Stratification factors are site (Oxford, London, Sussex), time since trauma (< 18 months/ ≥ 18 months), and PTSD symptom severity (PCL-5 score: high versus low). The emergency randomisation strategy is to randomly draw from 7 envelopes (3 iCT-PTSD, 3 iStress-PTSD, 1 wait). For patients taking psychotropic medication, randomisation takes place after they have been on a stable dose for 1 month. For those who are currently undergoing another psychological treatment, randomisation takes place after the end of this treatment.

Waitlist participants who have not recovered from PTSD at 13 weeks (end of waiting period) are randomised to either iCT or iStress at a 1:1 ratio using severity of PTSD symptoms and time since the trauma as stratification factors.

2.3. **Duration of the treatment/wait period**

iCT-PTSD and iStress-PTSD are delivered over a total of 26 weeks from randomisation. In the main treatment phase (13 weeks), patients complete online modules and are offered 12 weekly phone calls with therapist, as well as regular contact via messaging. In the second (booster) treatment phase (13 weeks), therapists continue to release treatment modules if needed and support the patient with up to 3 monthly phone calls and messages as needed. Patients retain access to the online programme for 1 year. They can also download the treatment modules they completed. The wait-list condition was set at 13 weeks to be comparable to the main treatment phase.

2.4. **Blinding**

Assessors of treatment outcome will be blinded. Therapists supporting the internet-based treatments, trial administrators and participants will not be blind to treatment allocation due to the nature of the intervention.

The trial statistician will be blind to the treatment condition represented by the grouping variable in the data file (blinding for wait-list is not possible because of the difference in allocation ratio). Unblinding of the trial statistician will occur after the initial draft of the statistical analysis report is signed off.

2.5. **Sample size calculations**

The trial has been powered to detect an effect size of Cohen's d = 0.50 for the comparison between iCT-PTSD and iStress-PTSD (see also section 3.2.). This effect size was chosen as it corresponds to clinically meaningful differences on the primary outcome measure (PCL-5), and was used in the NICE (2005) guidelines to define a clinically significant difference in placebo-controlled trials. For the comparison between psychological treatments and waitlists an effect size of d = 0.80 is considered as clinically meaningful by NICE (2005).

To detect a difference with an effect size of d = 0.50 with 80% power at α = .05, 63 participants per group are required. In addition, we have allowed for effects of clustering of observations within therapists, design factor = 1.18, assuming a conservative intra-class correlation of 0.01 (following Baldwin et al.'s (2011) recommendation) and an average cluster size of 12[1] and a coefficient of variation of CV= .68, and conservatively allowed for 15% for dropouts, yielding a sample size of 91 per group for this comparison (Eldridge, 2006). Thus, as the initial allocation ratio is 3:3:1 (iCT-PTSD: iStress-PTSD: Wait), a total of 217 participants will be randomly allocated (93 participants per treatment condition, and 31 for the waitlist). The power to detect a difference between the treatments and wait list conditions of d = 0.80 is greater than 98%. The total sample treated including post-wait allocations will therefore be up to 109 in one of the treatment arms and up to 108 in the other arm.

---

[1] The original power analysis had to be revised in November 2018 due to loss of therapists, and therapist absence due to illness and maternity leave, which increased the cluster size and coefficient of variation compared to the original power calculation.

2.6. Framework

The primary group comparison (hypotheses of highest priority) is whether iCT-PTSD is superior to iStress-PTSD. The primary outcome point is 13 weeks post randomisation.

Second, the efficacy of both treatments is also compared to the waitlist to account for possible natural recovery during the waiting period, i.e., to test whether iCT-PTSD is superior to waitlist and whether iStress-PTSD is superior to waitlist. There is no hierarchy in hypotheses for those two group comparisons.

2.7. End of the trial and data analysis

The end of the trial is defined at the time point when the last recruited patient completes the last follow-up period (long-term follow-up at 65 weeks).

No interim analyses are conducted.

The trial analysis is done after the end of the trial, as defined.

2.8. Data management

Anonymised data will be downloaded onto encrypted and password-secured computers at the Oxford Centre for Anxiety Disorders and Trauma. Any changes made to the data are stored in the audit log with a full history of changes being recorded.

3. General statistical analysis principles

3.1. Confidence intervals and p-values

The analysis of the primary research question (comparison in treatment efficacy between iCT-PTSD and iStress-PTSD) is carried out with a significance level of $p < .05$, two-sided. Analyses of whether each of the treatment arms is superior to waitlist is carried out at a significance level of $p < .025$ (0.05/2), two-sided.

Confidence intervals are reported on the 95% level.

3.2. Analysis population

Analyses will be intent-to-treat, using maximum likelihood estimates from all available data (see 5.2).

4. Trial population

4.1. Screening and eligibility

Participants must meet the following inclusion criteria:

1) Aged 18 and above.
2) Willing and able to provide informed consent.
3) Meets diagnostic criteria for PTSD as determined by the Structured Clinical Interview for DSM-5 [28].

4) Their current reexperiencing symptoms are linked to one or two discrete traumatic events that they experienced in adulthood or adolescence, or several traumatic episodes during a longer period of high threat (e.g., domestic abuse, war zone experiences).
5) PTSD is the main psychological problem needing treatment.
6) Able to read and write in English.
7) Access to the internet.
8) Willing to be randomly allocated to one of the psychological treatments or wait.
9) If taking psychotropic medication, the dose must be stable for at least 1 month before randomisation.
10) If currently receiving psychological therapy for PTSD, this treatment must have ended before randomisation

Exclusion Criteria

A person is not eligible if any of the following apply (assessed by clinician in the initial clinical assessment):

1) History of psychosis.
2) Current substance dependence.
3) Current borderline personality disorder.
4) Acute serious suicide risk.

Potential participants who are referred by the collaborating IAPT services, other NHS services or who self-refer will receive information about the trial on the telephone, answer basic questions about eligibility (e.g., age, access to internet), will be sent an information sheet and will have the possibility to ask questions. If they are interested, potential participants are invited for a clinical eligibility assessment by a clinical psychologist at one of the trial locations. Participants give written consent for this assessment.

If the clinical assessment shows that the participant is eligible for the trial, they will be informed, will have the opportunity to ask further questions and if they agree to participate, sign the informed consent form for the trial. Participants who are not eligible or do not wish to take part in the trial are advised about treatment options and signposted to appropriate services.

4.2. **Recruitment**

Participants will be recruited at three locations (Oxford: Oxford Centre for Anxiety Disorders and Trauma, University of Oxford; London: Institute of Psychiatry, Psychology and Neuroscience, King’s College London and Centre for Anxiety Disorders and Trauma, South London and Maudsley NHS Foundation Trust; Sussex: Sussex Partnership NHS Foundation Trust). Recruitment will be mainly via referral from collaborating IAPT services (Buckinghamshire, Berkshire, Croydon, Lambeth, Lewisham, Oxfordshire, Southwark, Brighton & Hove, and East Sussex). Referrals from GPs and other NHS services such as Hospital Trauma Services, local therapists and self-referrals are also accepted.

4.3. Withdrawal from treatment or follow-up or withdrawal of data

Dropout, or premature termination from the study or treatment at any point after randomisation, will be recorded along with reason for discontinuation or termination. Participants can choose to withdraw from the trial intervention, withdraw from follow-up, withdraw from both aspects, or withdraw from both aspects and ask that previously collected data not be used. Their care in the NHS will not be affected at any time by declining to participate or withdrawing from the trial. As much information as possible will be collected from protocol non-adherers including reasons for non-adherence. Participants' available data will be included in the analysis unless they ask for their previously collected data not to be used. Withdrawn participants are not replaced. If participants gave reasons for withdrawal, reasons are reported.

4.4. Descriptive statistics and patients' baseline characteristics

Available data for the total sample as well as the randomised groups (iCT-PTSD, iStress-PTSD, waitlist, post-wait iCT-PTSD, post-wait iStress) will be summarised in the form of means and standard deviations (or frequencies and percentages if categorical) for each outcome measure for each assessment point.

Demographic measures, such as sex, level of education, ethnicity, marital status, and clinical descriptors such as trauma type and comorbidity at baseline are reported for allocation groups and the total sample (frequencies and percentages for categorical data, means and standard deviations for numeric data). Occurrence of missing data for the randomised groups, for the drop-out and compliance groups, and the total sample are reported. No statistical comparisons of baseline data will be performed.

In addition, for comparison with other studies, we will present the percentage of patients meeting criteria for PTSD and recovery on the CAPS as well as IAPT recovery criteria by allocation for the assessments at 13, 26, 39 and 65 weeks. We will also report the percentage of patients meeting criteria for clinically significant change and deterioration on the PCL-5.

5. Trial analysis

5.1. Measures and outcome definitions

The assessment schedule is shown in Figure 1 (Appendix). The main assessment points are at baseline, 6 weeks, 13 weeks (post main treatment phase with weekly phone calls/wait; primary outcome time point), 26 weeks (post booster treatment phase with monthly phone calls, first follow-up), 39 weeks (second follow-up), and 65 weeks (long-term follow-up).

13 weeks is the primary outcome time point for the estimation of the treatment effects.

5.1.1. Primary outcome

PTSD symptom severity (PTSD checklist for DSM-5 (PCL-5); Weathers et al., 2015), assessed at baseline, weekly during the intervention/ 6 weeks for wait, 13 weeks (post treatment with weekly phone calls/wait; primary outcome time point), 26 weeks (post booster treatment phase with monthly phone calls, first follow-up), 39 weeks (second follow-up), and 65 weeks (long-term follow-up).

5.1.2. Secondary and further outcomes: symptom and well-being measures

5.1.2.1. Secondary outcomes: further PTSD symptom measures

Rater-assessed PTSD symptom severity assessed by trained independent raters (Clinician-Administered PTSD Scale for DSM-5 (CAPS-5); Weathers et al., 2015), assessed at baseline and at 13 weeks, 26 weeks, 39 weeks, and 65 weeks.

IAPT measure of PTSD symptom severity (Impact of Event Scale – Revised (IES-R); Weiss et al., 1996), assessed at baseline, weekly during the weekly phase of the intervention/ 6 weeks for wait, at 13 weeks, 26 weeks, 39 weeks, and 65 weeks. (This scale was included because it was the outcome measure used to assess PTSD in the participating IAPT services at the time of the study, it is needed to calculate the IAPT recovery criterion).

5.1.2.2. Further outcome measures: other clinical symptoms, disability, wellbeing and quality of life, sleep

Depression symptom severity (Patient Health Questionnaire (PHQ-9); Kroenke, Spitzer, & Williams, 2006), assessed at baseline, weekly during the weekly phase of the intervention/ 6 weeks for wait, at 13 weeks, 26 weeks, 39 weeks, and 65 weeks.

Anxiety symptom severity (Generalized Anxiety Disorder (GAD-7); Spitzer, Kroenke, Williams, & Löwe, 2006), assessed at baseline, weekly during the weekly phase of the intervention/ 6 weeks for wait, at 13 weeks, 26 weeks, 39 weeks, and 65 weeks.

Severity of disability (Work and Social Adjustment Scale (WSAS); Mundt, Marks, Shear, & Greist, 2002), assessed at baseline, weekly during the weekly phase of the intervention/ 6 weeks for wait, at 13 weeks, 26 weeks, 39 weeks, and 65 weeks.

Well-being, assessed by the WHO-5 Well-being Index (WHO, Topp et al., 2015) at baseline, 6 weeks, 13 weeks, 26 weeks, 39 weeks, and 65 weeks.

Endicott Quality of Life Scale QoL (Rapaport, Clary, Fayyad, & Endicott (2000), assessed at baseline, 6 weeks, 13 weeks, 26 weeks, 39 weeks, and 65 weeks.

Severity of problems with sleep (Insomnia Severity Index (ISI); Morin, Belleville, Bélanger, & Ivers, 2011), assessed at baseline, weekly during the weekly phase of the intervention/ 6 weeks for wait, at 13 weeks, 26 weeks, 39 weeks, and 65 weeks.

5.1.3. Treatment satisfaction and experience

The Online Treatment Experience Interview and IAPT Patient Experience Questionnaire is conducted at 13 weeks to assess treatment satisfaction and aspects of treatment experienced as helpful or unhelpful.

5.1.4. Health economic measures

Health economic measures (Euroqol EQ-5D-5L (Whynes & Group, 2009); iMTA Productivity Cost Questionnaire PCQ (Boumans, Krol, Severens, Koopmanschap, Brouwer, & Roijen, 2015); Endicott Quality of Life Scale QoL (Rapaport, Clary, Fayyad, & Endicott (2000); Client Service Receipt Inventory CSRI (Chisholm, Knapp, Knudsen, Amaddeo, Gaite, & van Wijngaarden, 2000); employment status and state benefits; assessed at baseline,

at 13 weeks, 26 weeks, and 39 weeks. Treatment delivery costs will be calculated from therapist records of the number of minutes per week therapists spent messaging and talking with the participant.

5.1.5. **Patient adherence measures**

For both iCT and iStress we will report means and standard deviations for:

1) Time spent by patients logged into the programme.
2) Number of therapist phone calls attended.

and number and proportions for

3) Randomised patients starting treatment.
4) Patients dropping out of treatment, and reasons if known.
5) Core modules completed.
6) Released modules completed.

5.1.6. **Process Measures**

Severity of negative appraisals about the traumatic event and/or its consequences (Posttraumatic Cognitions Inventory (PTCI); Foa, Ehlers, Clark, Tolin, & Orsillo, 1999; short version), assessed at baseline, weekly during the intervention/ at 6 weeks for wait-list, at 13 weeks, 26 weeks, and 39 weeks.

Trauma memory qualities (Trauma Memory Questionnaire (MQ); Halligan, Michael, Clark, & Ehlers, 2003; short version), assessed at baseline, weekly during the weekly phase of the intervention/ at 6 weeks for wait-list, at 13 weeks, 26 weeks, and 39 weeks.

Unhelpful responses to intrusive memories (Responses to Intrusions Questionnaire (RIQ, short version); Clohessy & Ehlers, 1999; Murray, Ehlers, & Mayou, 2002), assessed at baseline, weekly during the weekly phase of the intervention/ at 6 weeks for wait-list, at 13 weeks, 26 weeks, and 39 weeks.

Use of general safety behaviours (excessive precautions; Safety Behaviours Questionnaire (SBQ, short version); Dunmore, Clark, Ehlers, 1999), assessed at baseline, weekly during the weekly phase of the intervention/ waiting period, at 13 weeks, 26 weeks, and 39 weeks.

Severity of dissociation (State-Trait Dissociation Questionnaire (TSDQ); Murray, Ehlers, & Mayou, 2002; short version), assessed at baseline, weekly during the weekly phase of the intervention/ 6 weeks for wait, at 13 weeks, 26 weeks, and 39 weeks.

Self-efficacy (General Self Efficacy Scale (GSES); Schwarzer et al., 1995), assessed at baseline, 6 weeks, 13 weeks, and 39 weeks.

Therapeutic working alliance, patient and therapist (Working Alliance Inventory (WAI), Horvarth & Greenberg, 1989), assessed at weeks 2 and 6.

5.1.7. **Clinical Measures**

Alcohol use (Alcohol Use Disorders Identification Test (AUDIT); Babor et al., 2001), assessed at baseline, 6 weeks, 13 weeks, 26 weeks, 39 weeks, and 65 weeks.

Severity of complex PTSD symptoms as defined in ICD-11 (International Trauma Questionnaire; Hyland et al., 2017), assessed at baseline, 6 weeks, 13 weeks, 26 weeks, 39 weeks, and 65 weeks.

Emotions (Emotion Rating Scale), assessed at baseline, at week 6, at 13 weeks, 26 weeks, and 39 weeks.

5.1.8. **Treatment credibility**

Treatment credibility rated by patient (Credibility Rating; Borkovec & Nau, 1972) will be assessed at week 2.

5.1.9. **Therapists' adherence to treatment protocol and competence**

Therapists' adherence to treatment components will be assessed by independent raters from messages therapists send via the online system and randomly selected audio recordings of the phone calls between therapist and participant. Competence of delivery is assessed by an independent rater using an adapted version of the Cognitive Therapy Rating Scale – Revised (Blackburn et al., 2001).

5.1.10. **Adverse effect monitoring**

Adverse events monitoring, weekly during weekly treatment phase and monthly during the monthly booster treatment phase by therapist, and by independent assessors at 13 weeks, 26 weeks, 39 weeks, and at 65 weeks.

5.2. **Analysis methods**

5.2.1. **iCT-PTSD versus iStress-PTSD**

5.2.1.1. **Primary outcome measure**

Linear mixed effects modelling is used to test the primary, secondary, and further research questions on symptoms and further outcomes as specified. An unstructured covariance structure matrix is used. Separate models for the treatment (including booster period) effects and maintenance of treatment effects are analysed.

For the model capturing the treatment and booster period effects, both repeated assessments (fixed effects, level 1; week 6, week 13 = primary outcome time point, week 26) and treatment group (random intercepts for the patients on level 2) are specified as categorical variables for reasons of flexibility in the modelling approach. Both, main effects of treatment group, and time point and treatment group interactions are tested (all fixed effects). Superiority is shown by a significant main effect for treatment group or a significant interaction between time point and treatment group in favour of iCT-PTSD.

Covariates are the baseline stratification variables for randomisation and baseline levels of the respective outcome variables (see section 5.3).

Sensitivity analyses are specified in section 5.7.

Each therapist treated patients in both active treatment groups and patients were allocated to both treatments and waitlist at each site. Therapists are not nested within sites. Sites or

therapists are introduced as fixed effects into the models separately. If the primary analysis shows that therapists or sites do not have any effect on outcome, any effects of therapists or sites are not considered in the final model or any further models.

Maintenance of the hypothesised group difference is tested in the follow-up period. For the maintenance effect model linear mixed effects regression models with PCL-5 scores during follow-up as outcomes, both repeated assessments (posttreatment (26 weeks or if not available 13 weeks), week 39, week 65 = long term follow-up; fixed effects on level 1) and treatment condition (iCT-PTSD versus iStress-PTSD, patients' intercepts are allowed to vary randomly) as main effects and their interaction are specified as fixed effects in the analysis.

Baseline values of the outcome variable are included as covariate for the primary analysis (see section 5.3).

For handling of post-wait allocation data, see sensitivity analyses (see section 5.7).

5.2.1.2. **Secondary and further outcome measures**

Parallel analyses will be performed for the secondary measures of PTSD symptom severity (CAPS, IES-R) as well as further outcome measures: depression symptom severity, anxiety symptom severity, disability, wellbeing, quality of life, and sleep symptom severity.

5.2.2. **iCT-PTSD OR iStress-PTSD versus wait-list**

5.2.2.1. **Primary outcome measure**

To compare effects of each of the treatments with the waitlist condition on the PCL-5, linear mixed effects regression models with repeated assessments (categorical; week 6, week 13) and treatment condition (categorical; iCT-PTSD OR iStress-PTSD versus wait-list) and their interaction (all fixed effects with patients' intercepts allowed to vary randomly) are specified and analysed. Superiority is shown by a significant main effect for allocation group or a significant interaction between time point and allocation group in favour of iCT-PTSD or in favour of iStress-PTSD over waitlist.

5.2.2.2. **Secondary and further outcome measures**

Parallel analyses will be conducted for the CAPS (scores at 13 weeks) and the IES-R (6 weeks and 13 weeks) and for the other outcome measures.

5.2.3. **Health economic analyses**

The health economic analyses are conducted and reported by Dr. Tsiachristas and are therefore not part of this statistical analysis plan. A plan for the health economic analyses will be agreed.

5.2.4. **Mediators and moderators of therapeutic change**

If possible with regards to characteristics of missing data, a longitudinal structural equation modelling approach is used to test for any mediators of change (weekly process measures, see 2.4.6) in PTSD symptoms (PCL-5; baseline to week 13) during the treatment interval in the

iCT-PTSD condition. If not possible, linear mixed modelling with the Baron and Kenny approach for mediation is used. It is also analysed whether allocation to treatment group (iCT-PTSD versus iStress-PTSD) moderates this effect.

A linear regression approach is used to test whether therapeutic alliance (WAI) at week 2 and change between week 2 and 6 predict PTSD symptoms at 13 weeks (PCL-5), controlled for baseline PTSD symptoms.

Linear regression analyses are used to test whether change in self-efficacy from baseline to post-treatment at 13 weeks predicts PTSD symptom severity at 13 weeks (PCL-5; 13 weeks is primary outcome time point), controlled for baseline PTSD symptom severity. It is also analysed whether allocation to treatment group (iCT-PTSD versus iStress-PTSD) moderates this effect.

A range of potential covariates/moderators that might affect outcomes will be explored, such as gender, education level, ethnicity, age at main index trauma, trauma type, number of traumas, severity of physical consequences of the trauma, history of childhood trauma, complex PTSD symptoms, comorbid major depression, comorbid anxiety disorder, substance use and SAPAS score.

The analysis of mediators and predictors/ moderators of change is done with the whole sample, including post-wait allocation data.

5.2.5. **Experience with online treatment**

Means, standard deviations, and sample size for the IAPT Patient Experience Questionnaire and the quantitative questions in the Online Treatment Experience Interview at 13 weeks are reported.

Responses to the qualitative questions in the interview are thematically grouped according to their content, and frequencies are reported. Interrater reliability will be checked with 10% second ratings.

5.3. **Adjustment for covariates**

Baseline level of the respective outcome variable and stratification factors are included as covariates in the models for the primary, secondary, and further symptom and wellbeing outcomes with regards to the estimation of the treatment effects. Baseline level of the respective outcome variables are included as a covariate in the analyses of maintenance of treatment effects.

5.4. **Assumptions**

Assumptions for linear mixed effects modelling and structural equation modelling are tested. If violated, a resampling approach is used.

5.5. **Effect sizes**

Adjusted mean differences with 95% CI (two-sided) are reported for the treatment effects from results of mixed effects models. Adjusted mean differences between groups at each time point and within groups between each time point are reported. Cohen’s d is calculated by

dividing the treatment effect (= adjusted mean difference) by the standard deviation of the whole sample at baseline. As stated in the published trial protocol, Cohen's d is categorised as statistically significant, trend for superiority (nonsignificant effect size of $d \geq .25$), possible small superiority (nonsignificant effect size of $0.25 < d \geq .10$), and possible equivalence (nonsignificant effect size between $-0.10 < d > 0.10$).

5.6. Missing data

Missing data mechanisms are explored and reported: Frequencies and percentages of missing data and losses to follow-up are reported by randomised group. Missing data between the treatment arms and the waitlist group are compared and the results are reported.

The availability of the outcome data for the primary outcome measures and the primary time point is summarised per randomised group. Logistic regression models are used to explore any association between baseline data and the availability of the primary outcome data.

Sensitivity analyses are carried out to test robustness of the results to any missing data mechanism with regards to the primary outcome.

5.7. Sensitivity analyses

Sensitivity analyses are carried out whether there are differences in the primary outcome at the primary times point (PTSD symptom severity (PCL-5) at 13 weeks) between 1) analyses including cases with missing data versus 2) analyses including covariates predictive of missing data versus 3) imputation versus 4) analysis of the whole sample including post-wait allocation.

If the results for 4) are the same as for the primary analysis, post-wait allocation data will be included in further analyses of the two treatments such as the moderator and mediation analyses and analyses of other outcomes.

5.8. Subgroup analysis

No subgroup analyses are planned.

5.9. Additional analyses

5.9.1. Exploratory analysis: Trauma-focused use of exposure in iStress-PTSD

Therapists rate to what extent (percentage) participants used exposure in iStress-PTSD in a trauma-focused manner (e.g., systematic exposure to reminders of trauma or visiting the trauma site versus exposure other nontrauma-related anxiety-provoking situations). Linear regression models will test whether the degree of using iStress-PTSD exposure in a trauma-focussed manner has an effect on PCL-5 scores at 13 and 26 weeks, controlled for baseline PCL-5 scores.

5.9.2. **CACE analysis**

A complier-average causal effect analysis (CACE) is used to test the influence of treatment compliance on the treatment effect. Compliance with treatment is defined as per-protocol based on the minimum therapy to achieve benefits. This is operationalised as follows.

Patients in iCT-PTSD completed the following core procedures in the modules and/or phone calls:

- Treatment rationale and individual case formulation
- Reclaiming/rebuilding your life
- At least one of the core memory techniques: Updating memories and/or trigger discrimination

Patients in iCT-Stress completed the following core procedures through the modules and/or phone calls:

- Treatment rationale and psychoeducation about stress and PTSD
- In balance and activity planning
- Learned least one of the core stress management techniques (applied relaxation, thought challenging, mindfulness, sleep efficiency)

CACE is then compared with the primary analysis and the results are reported.

5.10. **Safety analysis and monitoring for potential negative effects of therapy**

The standard definitions for adverse events (AE) and serious adverse events (SAE) are used and detailed in the trial protocol.

AE, SAE, and self-harm and harm to others were monitored throughout the treatment phase (during weekly and monthly phone calls with therapist) and follow-ups (independent assessments). Any AE or SAE was evaluated by the principal investigator or another qualified clinical psychologist from the trial team in terms of any possible relationship to the treatment and expectedness. The only expected side effect of both treatment arms, of which participants were informed in the information sheet, is a short-term increase in distress due to remembering the traumatic event and/or facing trauma reminders.

The total number of treatment-related and unrelated AE and SAE and the number of SAE per patient (if at least one SAE) are reported for each randomised group. A comparison between the treatment arms regarding the total number of treatment-related and unrelated AE and SAE and the number of SAE per patient (if at least one) is reported. The safety analysis is also ITT. Results of the statistical tests and ratios are reported. If there are any differences between the allocation groups, additional analyses will be performed to investigate whether those might have led to heterogeneity in treatment effects.

Independent assessors reported that the Covid pandemic had treatment-unrelated negative effects on some of the patients. It is therefore compared between the allocation groups whether there are any differences with regards to a treatment-unrelated adverse effect due to the Covid pandemic. If there are any differences between the allocation groups, additional analyses will be performed to investigate whether those might have led to heterogeneity in treatment effects.

In line with a recent consensus statement on assessing negative effects of internet interventions, we will assess patients' evaluation of the internet modules and design through a feedback sheet at the end of each module, comments made to therapists in weekly phone

calls and the Online Treatment Experience Interview at 13 weeks. We will also determine the percentage of patients who experience symptom deterioration after therapy/wait-list, i.e. a statistically reliable change in an unfavourable direction on the PCL-5 or CAPS-5. Results of the statistical tests and ratios are reported.

5.11. Software

All analyses are done in RStudio (R Studio Team, most current version by the time of trial analysis will be used and reported in publication) within the R environment (R Core Team, most current version by the time of trial analysis will be used and reported in publication). The R packages *nlme* (Bates et al., 2015) and *lme4* (Pinheiro et al., 2020) will be used for the main trial analyses and the package *lavaan* (Rosseel, 2012) will be used for analysing the mediation research questions using structural equation modelling.

6. References

Babor TF, Higgins-Biddle JC, Saunders JB, Monteiro MG. The Alcohol Use Disorders Identification Test: guidelines for use in primary care. 2nd ed. Geneva: Department of Mental Health and Substance Dependence. World Health Organization; 2001.

Baldwin, S. A., Murray, D. M., Shadish, W. R., Pals, S. L., Holland, J. M., Abramowitz, J. S., et al. (2011). Intraclass correlation associated with therapists: estimates and applications in planning psychotherapy research. Cognitive Behaviour Therapy, 40(1), 15-33.

Bates, D., et al. (2015). Fitting linear mixed effects models using lme4. *Journal of Statistical Software, 67*, 1-48.

Bisson JI, Roberts NP, Andrew M, Cooper R, Lewis C. Psychological therapies for chronic post-traumatic stress disorder (PTSD) in adults. Cochrane Database of Systematic Reviews 2013, Issue 12. Art. No.: CD003388. DOI: 10.1002/14651858.CD003388.pub4.

Blackburn IM, James IA, Milne DL, Baker C, Standart S, Garland A, Reichelt FK. The Revised Cognitive Therapy Scale (CTS-R): psychometric properties. Behav Cogn Psychother. 2001;29(4):431–46.

Borkovec TD, Nau SD. Credibility of analogue therapy rationales. J Behav Ther Exp Psychiatry. 1972;3:257–60.

Bouwmans C, Krol M, Severens H, Koopmanschap M, Brouwer W, Roijen LH. The iMTA Productivity Cost Questionnaire: A Standardized Instrument for Measuring and Valuing Health- Related Productivity Losses. Value Health. 2015;18(6):753-758.

Chisholm D, Knapp MR, Knudsen HC, Amaddeo F, Gaite L, van Wijngaarden B. Client Socio- Demographic and Service Receipt Inventory--European Version: development of an instrument for international research. EPSILON Study 5. European Psychiatric Services: Inputs Linked to Outcome Domains and Needs. Br J Psychiatry Suppl. 2000(39):s28-33.

Clohessy, S. & Ehlers, A. (1999). PTSD symptoms, response to intrusive memories, and coping in ambulance service workers. British Journal of Clinical Psychology, 38, 251-265.

Dunmore, E., Clark, D.M., & Ehlers, A. (1999). Cognitive factors involved in the onset and maintenance of PTSD. Behaviour Research and Therapy, 37, 809-829.

Foa, E.B., Ehlers, A., Clark, D.M., Tolin, D., & Orsillo, S. (1999). The Post-traumatic Cognitions Inventory (PTCI). Development and validation. Psychological Assessment, 11, 303-314.

Halligan, S. L., Michael, T., Clark, D.M., & Ehlers, A. (2003). Posttraumatic stress disorder following assault: the role of cognitive processing, trauma memory, and appraisals. Journal of Consulting and Clinical Psychology, 71, 419-431.

Horvath AO, Greenberg LS. Development and validation of the Working Alliance Inventory. Journal of Counseling Psychology, Vol 36(2), Apr 1989, 223-233. http://dx.doi.org/10.1037/0022-0167.36.2.223

Hyland P, Shevlin M, Brewin CR, Cloitre M, Downes AJ, Jumbe S, et al. Validation of posttraumatic stress disorder (PTSD) and complex PTSD using the International Trauma Questionnaire. Acta Psychiatr Scand. 2017;136:313–22.

Kroenke K, Spitzer RL, Williams JBW. The PHQ-9. Journal of General Internal Medicine. 2001;16(9):606-613.

Morin CM, Belleville G, Bélanger L, Ivers H. The Insomnia Severity Index: psychometric indicators to detect insomnia cases and evaluate treatment response. Sleep. 2011;34(5):601-8.

Mundt JC, Marks IM, Shear MK, Greist JH. The work and social adjustment scale: a simple measure of impairment in functioning. British Journal of Psychiatry. 2002;180:461-464.

Murray, J., Ehlers, A. & Mayou, R.A. (2002). Dissociation and posttraumatic stress disorder: Two prospective studies of road traffic accident victims. British Journal of Psychiatry, 180, 363-368.

National Institute for Health and Care Excellence. Post-traumatic stress disorder (PTSD): The management of PTSD in adults and children in primary and secondary care. London: NICE. 2005. (Clinical Guideline 26).

National Institute for Health and Care Excellence. Posttraumatic stress disorder (PTSD). London: NICE. 2018. (Clinical Guideline 116). Available from: https://www.nice.org.uk/guidance/ng116

Persson Asplund, R., Dagöö, J., Fjellström, I., Niemi, L., Hansson, K., Zeraati, F., Ziuzina, M., Ljótsson, B., Carlbring, P., & Andersson, G. (2017). Internet-based cognitive behavioral stress management and feedback training for managers: A randomized controlled trial. Manuscript under review.

Pinheiro, J., et al. (2020). *nlme: Linear and nonlinear mixed effects models* 2020. https://CRAN.R-project.org/package=nlme.

Rapaport MH, Clary C, Fayyad R, Endicott J. Quality-of-life impairment in depressive and anxiety disorders. Am J Psychiatry. 2005;162(6):1171-1178.

Rosseel Y (2012) lavaan: An R package for structural equation modeling. Journal of Statistical Software 48(2), 1–36.

Rozental A, Andersson G, Boettcher J, Ebert D, Cuijpers P, Knaevelsrud C, et al. Consensus statement on defining and measuring negative effects of Internet interventions. Internet Interv. 2014;1: 12-19.

RStudio Team (most current version at time of trial analysis) RStudio: Integrated development for R. Boston, MA: RStudio, Inc.

R Core Team (most current version at time of trial analysis). R: A language and environment for statistical computing. R Foundation for Statistical Computing, Vienna, Austria. R Version 3.6.1

Sachschal J, Woodward, E, Wichelmann JM, Haag K and Ehlers A (2019). Differential effects of poor recall and memory disjointedness on trauma symptoms. Clinical Psychological Science, 1-10.

Schwarzer R, Jerusalem M. Generalized Self-Efficacy scale. In: Weinman J, Wright S, Johnston M, editors. Measures in health psychology: a user's portfolio. Causal and control beliefs. Windsor: NFER-NELSON; 1995. p. 35–7.

Schwarzer, R., & Jerusalem, M. (1995). Generalized Self-Efficacy scale. In J. Weinman, S. Wright, & M. Johnston, Measures in health psychology: A user's portfolio. Causal and control beliefs (pp. 35-37). Windsor, UK: NFER-NELSON.

Spitzer Rl, Kroenke K, Williams JW, Löwe B. A brief measure for assessing generalized anxiety disorder: The GAD-7. Archives of Internal Medicine. 2006;166(10):1092-1097

Van Buuren, S. & Groothuis-Oudshoorn, K. (2011). mice: Multivariate Imputation by Chained Equations in R. Journal of Statistical Software, 45(3), 1-67.

Weathers FW, Blake DD, Schnurr PP, Kaloupek DG, Marx BP, Keane TM. (2015). The Clinician-Administered PTSD Scale for DSM-5 (CAPS-5). Scale available from the National Center for PTSD at www.ptsd.va.gov.

Weathers FW, Litz BT, Keane TM, Palmieri PA, Marx BP, Schnurr PP. The PTSD Checklist for DSM-5 (PCL-5). Scale available from the National Center for PTSD at www.ptsd.va.gov.

Weiss DS, Marmar CR. The Impact of Event Scale–Revised. In: Wilson J, Keane TM, editors. Assessing psychological trauma and PTSD. New York: Guilford; 1996. p. 399–411.

Whynes DK, Group T. Responsiveness of the EQ-5D to HADS-identified anxiety and depression. J Eval Clin Pract. 2009;15(5):820-825.

Wild, J., Warnock-Parkes, E., Grey, N., Stott, R., Wiedemann, M., Canvin, L., Rankin, H., Shepherd, E., Forkert, A., Clark, D.M, & Ehlers, A. (2016). Internet-delivered cognitive therapy for PTSD: a development pilot series, European Journal of Psychotraumatology. 7: 31019. DOI: http://dx.doi.org/10.3402/ejpt.v7.31019

Zetterqvist, K., Maanmies, J., Ström, L. & Andersson, G. (2003) Randomized controlled trial of internet-based stress management. Cognitive Behaviour Therapy, 32, 151-160.

7. Appendices

Figure 1: Schedule for Enrolment, Interventions, and Assessments

	STUDY PERIOD							
	Enrolment	Allocation and Baseline Assessment	Post-allocation					Long-term follow-up
TIMEPOINT			During Treatment	6 weeks	13 weeks	26 weeks	39 weeks	65 weeks
**Eligibility assessment**								
Informed consent for eligibility assessment	X							
Demographics (Patient Registration Form)	X							
Structured Clinical Interview for DSM-5 (SCID-5)	X							
Life Event Checklist (LEC)	X							
Psychiatric Diagnostic Screening Questionnaire (PDSQ)	X							
Standardized Assessment of Personality Abbreviated Scale (SAPAS)	X							
Structured Clinical Interview for DSM-5 Screening Personality Questionnaire (SCID5-SPQ) and Structured	X							

Clinical Interview for DSM-5 Personality Disorder, borderline and paranoid personality disorder only								
SLAM Risk Screen and PCMIS Screener Clinical Interviews	X							
Medication use	X	X			X	X	X	X
Informed consent for assessment / trial	X	(X)*						
Randomisation		X						
**INTERVENTIONS**<br><br>Internet-delivered cognitive therapy for PTSD (iCT-PTSD)				Main treatment		booster		
Internet-delivered stress management therapy for PTSD (iStress-PTSD)				Main treatment		booster		
Wait-list condition					**			

**ASSESSMENTS**<br><br>**Outcomes: Symptom and Well-Being Measures**								
PTSD Checklist for DSM 5 (PCL-5)	X	X	X***	X	X	X	X	X
Clinician Administered PTSD Scale for DSM 5 (CAPS-5)		X			X	X	X	X
Impact of Event Scale revised	X	X	X***	X	X	X	X	X
Patient Health Questionnaire (PHQ-9)	X	X	X***	X	X	X	X	X
Generalized Anxiety Disorder Scale 7-items (GAD-7)	X	X	X***	X	X	X	X	X
WHO(Five) Well-Being Index		X		X	X	X	X	X
Work and Social Adjustment Scale (WSAS)	X	X	X***	X	X	X	X	X
Endicott Quality of Life Scale (QoL)		X		X	X	X	X	X
Insomnia Sleep Index (ISI)		X	X***	X	X	X	X	X

**Health Economic Measures**								
Euroqol EQ-5D-5L12		X			X	X	X	
iMTA Productivity Cost Questionnaire (PCQ)		X			X	X	X	
Client Service Receipt Inventory (CSRI)		X			X	X	X	
Employment status and state benefits		X			X	X	X	
Treatment delivery costs (number of minutes therapist spent messaging and talking with patient).			X					
**Process Measures**								
Posttraumatic Cognitions Inventory (PTCI)		X	X***	X	X	X	X	
Trauma Memory Questionnaire (MQ)		X	X***	X	X	X	X	
Response to Intrusion Questionnaire(RIQ)		X	X***	X	X	X	X	
Safety Behaviours Questionnaire(SBQ)		X	X***	X	X	X	X	
Trait-State Dissociation Questionnaire(TSDQ)		X	X***	X	X	X	X	
Generalized Self Efficacy Scale(GSES)		X		X	X	X	X	
Online Treatment Experience Interview and IAPT Patient Experience Questionnaire					X			
Working Alliance Inventory (WAI)			2 weeks	X				

**Clinical Measures**								
Alcohol Use Disorders Identification Test (AUDIT)	X			X	X	X	X	X
International Trauma Questionnaire (complex PTSD)		X		X	X	X	X	X
Emotion scale		X		X	X	X	X	
Treatment Credibility			2 weeks					
Adverse event/effect monitoring			X	X	X	X	X	X
Medication use		X			X	X	X	X

* If ICF part 2 is not completed at the time of the eligibility assessment it will be completed at the beginning of the Baseline visit ahead of randomisation.

** followed by random allocation to iCT or iStress if still meets criteria for PTSD

*** collected by the online therapy programme, Improving Access to Psychological Therapies Services require weekly measures of IES-R, PHQ-9, GAD-7 and WSAS for patient records
